# Characterization of a Prawn OA/TA Receptor in *Xenopus* Oocytes Suggests Functional Selectivity between Octopamine and Tyramine

**DOI:** 10.1371/journal.pone.0111314

**Published:** 2014-10-28

**Authors:** Sami H. Jezzini, Dalynés Reyes-Colón, María A. Sosa

**Affiliations:** 1 Department of Anatomy & Neurobiology, School of Medicine, Medical Sciences Campus, University of Puerto Rico, San Juan, Puerto Rico, United States of America; 2 Institute of Neurobiology, Medical Sciences Campus, University of Puerto Rico, San Juan, Puerto Rico, United States of America; 3 Department of Biology, Arecibo Campus, University of Puerto Rico, San Juan, Puerto Rico, United States of America; University of Missouri, United States of America

## Abstract

Here we report the characterization of an octopamine/tyramine (OA/TA or TyrR1) receptor (OA/TA_Mac_) cloned from the freshwater prawn, *Macrobrachium rosenbergii*, an animal used in the study of agonistic social behavior. The invertebrate OA/TA receptors are seven trans-membrane domain G-protein coupled receptors that are related to vertebrate adrenergic receptors. Behavioral studies in arthropods indicate that octopaminergic signaling systems modulate fight or flight behaviors with octopamine and/or tyramine functioning in a similar way to the adrenalins in vertebrate systems. Despite the importance of octopamine signaling in behavioral studies of decapod crustaceans there are no functional data available for any of their octopamine or tyramine receptors. We expressed OA/TA_Mac_ in *Xenopus* oocytes where agonist-evoked trans-membrane currents were used as readouts of receptor activity. The currents were most effectively evoked by tyramine but were also evoked by octopamine and dopamine. They were effectively blocked by yohimbine. The electrophysiological approach we used enabled the continuous observation of complex dynamics over time. Using voltage steps, we were able to simultaneously resolve two types of endogenous currents that are affected over different time scales. At higher concentrations we observe that octopamine and tyramine can produce different and opposing effects on both of these currents, presumably through the activity of the single expressed receptor type. The pharmacological profile and apparent functional-selectivity are consistent with properties first observed in the OA/TA receptor from the insect *Drosophila melanogaster*. As the first functional data reported for any crustacean OA/TA receptor, these results suggest that functional-selectivity between tyramine and octopamine is a feature of this receptor type that may be conserved among arthropods.

## Introduction

**Figure 1:**
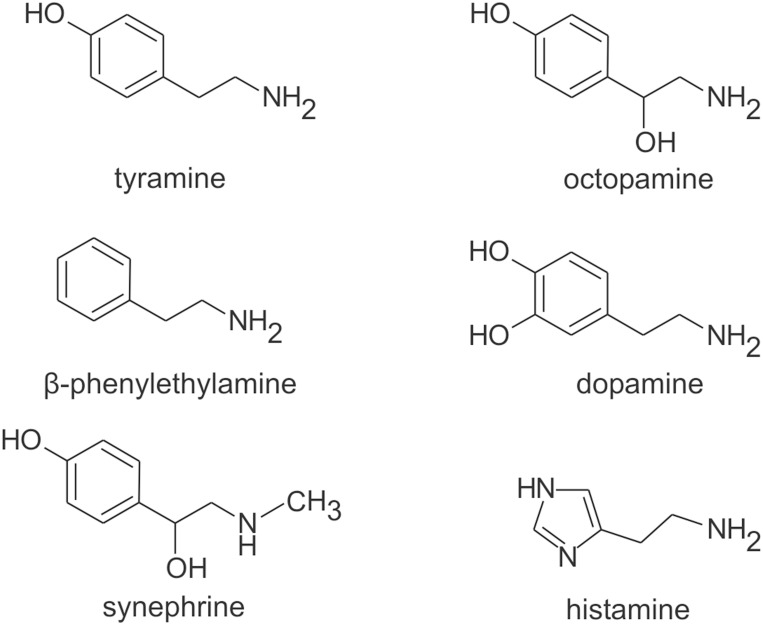
Aminergic agonists tested on OA/TA_Mac_. The biogenic amines are structurally similar small molecules. Tyramine, octopamine, dopamine, and histamine are naturally occurring *bona fide* neurotransmitters in invertebrates. In vertebrates β-phenylethylamine is a trace amine and synephrine is a synthetic adrenergic receptor agonist.

Octopamine, tyramine, and dopamine are structurally similar biogenic amines that are derived from the amino acid tyrosine (Fig. 1) [1]. These small signaling molecules have broad ranging cellular effects that are mediated by diverse and functionally complex receptor families. These include both ionotropic [2] and seven trans-membrane domain G protein-coupled receptors (GPCRs). The aminergic GPCRs have identified homologs in all major phyla including arthropoda [3]–[6]. In arthropods aminergic signaling is an important modulator of agonistic encounters and aggression [7]–[10] where the biogenic amines are known to function as both hormones and neurotransmitters [11]–[13]. We are currently cloning and characterizing aminergic GPCRs from the giant tropical prawn *Macrobrachium rosenbergii* as a step toward elucidating the molecular mechanisms associated with the formation and maintenance of prawn social hierarchies [14], [15].

Octopamine and tyramine [16] are endogenous signaling molecules that appear to have differential and sometimes antagonistic effects on physiology and behavior [17]–[20]. Furthermore, they are considered to serve homologous functions in invertebrates as do norepinephrine and epinephrine in mammals [4], [21], [22]. Close homology between the vertebrate adrenergic GPCRs and the invertebrate octopaminergic GPCRs supports this view [6]. In fact, insect octopamine receptors have been classified as α–adrenergic-like and ß-adrenergic-like based on comparisons with vertebrate receptors [4]. The tyramine receptors (TyrR) are also homologous to vertebrate α-adrenergic receptors but are distinguished from octopamine receptors in that they are more sensitive to tyramine [4]. Three groups of TyrR receptors have been so far identified. Members of the original group are designated TyrR1 [6] and are also referred to as OA/TA-type because they are sensitive to octopamine and tyramine [4]. The TyrR2 receptors identified in *Drosophila* are sensitive to tyramine but are not activated, or are weakly activated, by octopamine [23]–[25]. Most recently the TyrR3 group that responds to multiple biogenic amines has been identified, also in *Drosophila* [23].

Despite the large amount of behavioral data related to octopamine in crustaceans, and the amenability of crustacean nervous preparations to experimental analysis, few crustacean octopamine receptors have been cloned and characterized [26]. We previously reported the first cloned member of the OA/TA-type (TyrR1) GPCRs from decapod crustaceans, OA/TA_Mac_, cloned from the CNS of the freshwater prawn [14]. In this paper we report its functional characterization.

Aminergic receptors tend to exert complex intracellular effects through multiple signaling pathways. The α-adrenergic-like octopamine receptors typically cause increases in both intracellular calcium and cAMP [27]–[30]. The OA/TA receptors typically activate intracellular pathways that increase intracellular calcium and/or suppress cAMP levels [25], [31]–[35]. In addition to effects on multiple signaling pathways, “functional selectivity”, also known as “agonist-selective coupling”or “biased agonism”, is a property often observed among adrenergic-type receptors in both vertebrates [36], [37] and invertebrates. Functional selectivity refers to a measurable cellular effect appearing to be preferentially (or selectively) induced by different ligands acting through a single receptor [36], [38]–[42]. While it is often indicated by differential effects on measured levels of second messengers, functional selectivity is an operational term that can be inferred from any receptor dependent cellular output [42] such as desensitization or modulation of ionic currents.

Functional selectivity between octopamine and tyramine was observed for the first cloned OA/TA receptor (CG7485) [43], which was cloned from *Drosophila* [31], [35]. When CG7485 was expressed in Chinese Hamster Ovary (CHO) cells, octopamine more effectively increased intracellular calcium while tyramine more effectively reduced cAMP levels [43]. Functional selectivity was indicated by the fact that which transmitter displayed the greatest efficacy was dependent on which output (second messenger level) was considered. In addition to functional selectivity evoked through different agonists, concentration-sensitive effects caused by high and low concentrations of the same agonist have been observed for a number of insect octopamine receptors [27], [30], [44]. Functional selectivity arising from a concentration-sensitive response has been described for the α-adrenergic-like octopamine receptor CsOA1 (JN641302) [45]. High and low octopamine concentrations produced opposite behavioral effects on caterpillar hemocyte spreading and phagocytosis. In these immune cells that express CsOA1 endogenously, low concentrations of octopamine induced an increase in intracellular calcium, while high concentrations of octopamine induced both calcium and cAMP [45]. Thus, for CsOA1 functional selectivity was indicated both in terms of a behavioral effect (suppression or facilitation of cell motility) and an induction of an additional cellular process (cAMP production). While agonist-selective effects and concentration-sensitive effects are clearly documented for arthropod α-adrenergic-like GPCRs, directly opposing effects between tyramine and octopamine have not been reported.

Here we describe the first functional characterization of a crustacean tyramine/octopamine receptor, the prawn OA/TA_Mac_ [14]. The sensitivity of OA/TA_Mac_ to tyramine, octopamine and dopamine, as well as its pharmacological profile, are consistent with OA/TA type receptors from insects. OA/TA_Mac_ also appears to exhibit concentration-sensitive functional selectivity between tyramine and octopamine. The functional selectivity described here is observable as differential modulation of two different types of native currents of the *Xenopus laevis* oocyte. One is the direct calcium-dependent chloride current (we denote I_D_), evoked by transmitter application at a constant holding potential [46]. The second is the transient calcium-dependent chloride current (I_Cl-T_) evoked by step changes in voltage [47]. At high concentrations, tyramine evokes an additional intracellular process that results in a distinctly different I_D_ wave-form from that of octopamine. In addition, at high concentrations, tyramine increases, whereas octopamine decreases I_Cl-T_.

This is the first report of opposing cellular effects between tyramine and octopamine observed following the expression of an OA/TA receptor. This finding on the crustacean receptor agrees with previous findings from the insect OA/TA receptor (CG7485) that suggest the apparently minor chemical modification (a single hydroxyl group) between tyramine and octopamine (Fig. 1) can cause altered function of the OA/TA receptor [43]. Thus, these data provide evidence that functional selectivity between octopamine and tyramine may be a conserved property of arthropod OA/TA receptors.

## Results and Discussion

We used *Xenopus* oocytes to express the prawn OA/TA_Mac_ receptor by injection of synthetic cRNA. The injection of foreign RNA into *Xenopus* oocytes typically induces the expression of multiple native chloride and potassium channels [47]. Ligand evoked currents resulting from modulation of these channels have been used extensively in the characterization of heterologously expressed GPCRs [48]. Oocytes are typically voltage clamped at a constant membrane potential around −60 mV and deviations in holding potential are measured. We refer to currents measured in this way as direct-currents (I_D_) (after [46]) in order to distinguish them from the voltage-evoked transient current (I_Cl-T_) described later.

### Tyramine, octopamine and dopamine evoke complex direct-currents (I_D_) in OA/TA_Mac_ injected oocytes

**Figure 2:**
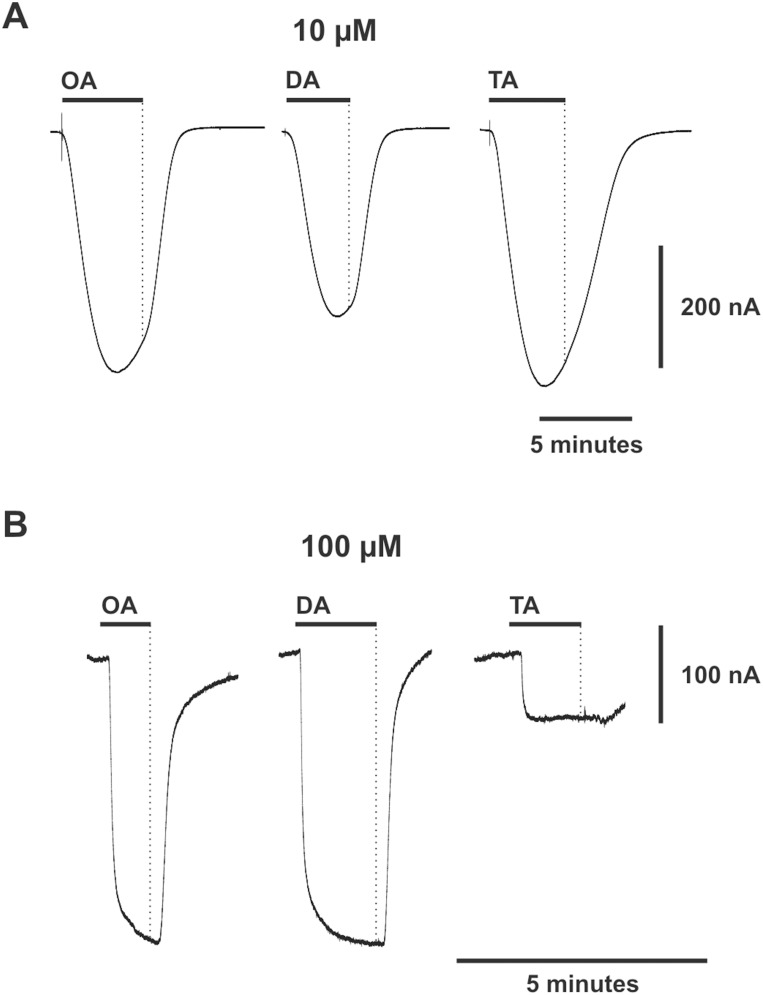
Octopamine (OA), dopamine (DA), and tyramine (TA) evoke complex currents in oocytes injected with OA/TA_Mac_ cRNA. The response is complex because the relative amplitudes change with concentration and apparent equilibrium is not reached for all concentrations. (**A and B**) Representative responses to prolonged applications at lower (10 µM) and higher (100 µM) concentrations. (**A**) TA evokes the greatest amplitude response at 10 µM. The responses never reach equilibrium. Instead, they begin to decline prior to the removal of agonist (end of black bar). (**B**) At 100 µM the currents appear to approach a plateau. The TA response plateaus more quickly causing its amplitude to be small relative to OA or DA. Oocytes were voltage clamped at −60 mV. Each of the three transmitters were applied to the same single oocytes using focal application via a triple-barrel pipette. Oocytes were co-injected with OA/TA_Mac_ receptor and CFTR cRNA. (Also see Fig. 6B for another example of long octopamine application).

We first tested receptor-injected oocytes for the occurrence of a response to biogenic amines. Applications of tyramine, octopamine, or dopamine produced an inward I_D,_ typically ranging between 50 nA and 500 nA, under a constant holding potential of −60 mV. These currents exhibited complex characteristics. The amplitude of tyramine evoked I_D_ at concentrations below 10 µM was greater than octopamine or dopamine within the same oocyte (Figs. 2A and S2). Under prolonged applications (on the order of minutes), at 10 µM, I_D_ did not consistently reach a stable steady state (Fig. 2A). Instead it would rise and then begin to decline prior to the removal of agonist (dotted line). However, at high concentrations, above 100 µM, I_D_ appeared to approach a stable plateau (Fig. 2B). In the case of tyramine, the plateau had an abrupt onset causing the amplitude of the response to be smaller than octopamine or dopamine within the same oocyte (Fig. 2B). Uninjected oocytes showed no response to any of these three compounds (n = 5, Fig. S1).

The fact that the tyramine response is relatively small at high concentration and relatively large at low concentration indicates that the current amplitude is a complex function of receptor activity. The interpretation of the high concentration plateau as an equilibrium state is difficult to reconcile with the failure of the response to exhibit an equilibrium state at low concentration. Consequently, the relative amplitudes of the evoked currents do not appear to be in direct proportion to the fractional activation of the receptor population. Therefore, in order to compare the effects of different compounds and concentrations on I_D_, we measured the sub-maximal dynamic response to a pulse of agonist, which was found to produce a reliable and repeatable measure. Both flow rate and pulse duration were precisely controlled and comparisons were made relative to currents within single oocytes.

### The dose dependence of amine-evoked direct-currents (I_D_)

**Figure 3:**
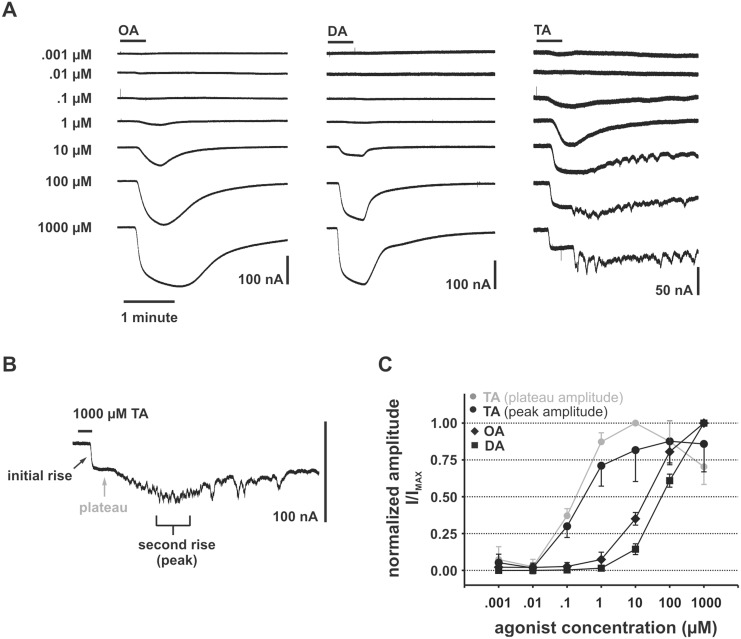
The dose dependence of transmitter evoked direct-current (I_D_) in oocytes expressing the OA/TA_Mac_ GPCR. (**A**) Representative responses to concentration series in three different oocytes injected with OA/TA_Mac_ using a 30 second perfusion-switch. Oocytes were voltage clamped at −60 mV. Octopamine (OA) and dopamine (DA) consistently evoked currents that increased in amplitude up to the maximum concentration. In contrast, currents evoked by tyramine (TA) increased in amplitude up to around 10 µM. TA-evoked responses also exhibited a more complex waveform at high-concentration. In some oocytes TA evoked oscillations that became more pronounced with increasing concentration. (**B**) The salient features of the high-concentration TA-evoked response are labeled. These include the abrupt appearance of a plateau during agonist application (black bar) followed by a second rise occurring during washout. In some oocytes oscillations appeared during recovery that were never seen with octopamine or dopamine. (**C**) The mean amplitudes of normalized currents (I/I_max_) recorded as in A are plotted against the concentration of agonist in µM. For TA, values measured and normalized at both the peak (black) and plateau (grey) are shown. Approximate EC_50_ values estimated from the plot are TA (EC_50_≈0.2 µM), OA (EC_50_≈21 µM), and DA (EC_50_≈63 µM). Error bars represent standard deviation of the normalized values and are shown in one direction for TA for clarity. Oocytes were injected with OA/TA_Mac_ receptor cRNA only (TA n = 4 oocytes; OA n = 6 oocytes; DA n = 5 oocytes).

To characterize the dose dependence of I_D_, series of single transmitters were applied to different individual oocytes injected with OA/TA_Mac_. Oocytes were voltage clamped at −60 mV and tyramine, (+/−)-octopamine, or dopamine were applied using 30 second perfusion-switched applications (see methods for more details). Octopamine and dopamine began to evoke visible inward currents at around 1 µM (Fig. 3). The amplitude of these currents continued to increase with increasing concentration up to 1000 µM. Currents evoked by tyramine became apparent at around 0.1 µM. However, the response to tyramine reached maximum amplitude at around 10 µM and remained comparable in size to previous applications as the concentration was increased (Fig. 3).

The relatively small amplitude of the tyramine response at high concentration coincided with a change in waveform that was clearly different from that evoked by octopamine or dopamine. Figure 3B shows the components of the tyramine response in detail. The black bar indicates the application of 1000 µM tyramine. There is an onset delay of which 6–7 seconds is due to the dead volume between the perfusion manifold and the oocyte chamber. Following the onset of the initial rise, the amplitude is limited by the abrupt appearance of a plateau. This amplitude-limiting plateau was invariably seen at high tyramine concentrations and never seen upon application of any other agonist we tested (up to concentrations of 1000 µM). During recovery a second rise becomes apparent that was often seen to coincide with minor oscillations. On occasion the oscillations were pronounced as shown in Figure 3A.

Amplitudes from currents recorded as shown in Figure 3A were normalized and plotted as dose-response curves in 3C. Curves normalized against both the plateau amplitude (grey) and peak amplitude (black) are shown for tyramine. The curves show that OA/TA_Mac_ injected oocytes were more sensitive to tyramine (EC_50_≈0.2 µM, n = 4) than octopamine (EC_50_≈21 µM, n = 6) or dopamine (EC_50_≈63 µM, n = 5). The tyramine curve for the peak amplitude is more variable (as indicated by the larger standard deviations) than the curve plotted for the plateau amplitude due to variability in the appearance of oscillations at the second rise. In addition, the grey curve has a maximum at 10 µM indicating that the relative amplitude of the plateau within an oocyte becomes smaller at concentrations above approximately 10 µM.

### The limited amplitude of the tyramine response coincides with the induction of an additional cellular process

**Figure 4:**
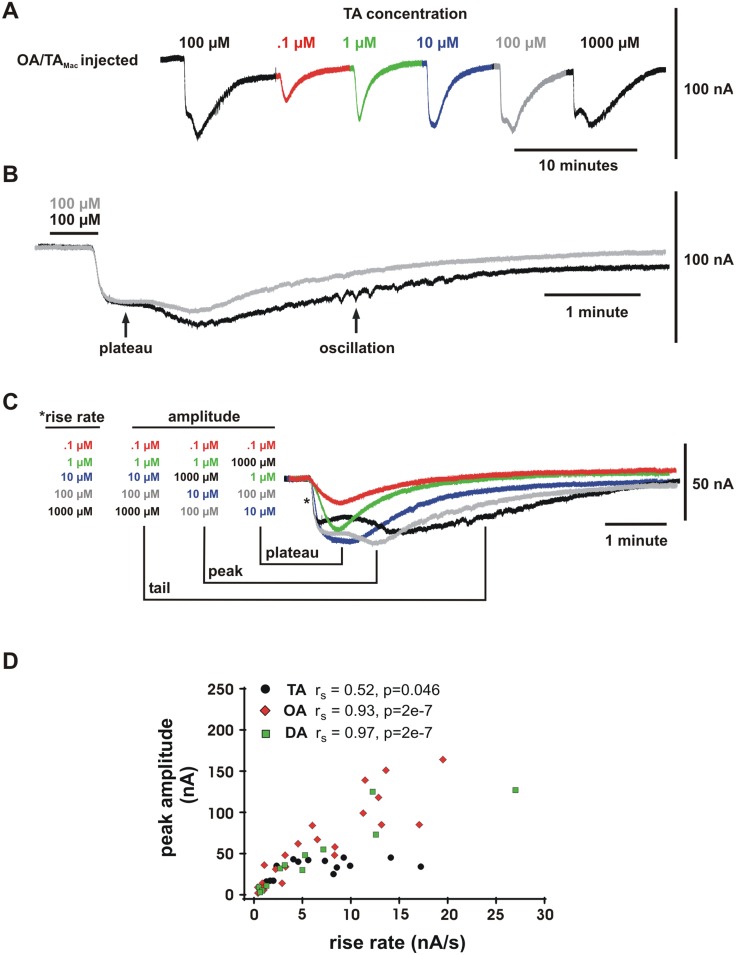
Tyramine (TA) selectively evokes an intracellular process that limits the response amplitude. (**A**) A concentration series applied to a single oocyte. Color-coding corresponds to the same responses examined in more detail in B and C. (**B**) The low amplitude of the TA response was not due to cumulative buildup of desensitization because it could be observed upon the first high-concentration application. An initial 100 µM TA response (black) is aligned by the stimulus application (black bar) with a second 100 µM response (grey). The plateau amplitude changes very little between the first and second response indicating that the small size is not a function of repetitive application. Desensitization of the second response is apparent in the tail as a reduction in amplitude, and as a loss of minor oscillation. (**C**) The plateau limits he amplitude of the TA response. Responses from A (color coded from 0.1 µM to 1000 µM) are aligned by the response onset and overlaid. The rank-order of rise rate at the initial rise (asterisk) and amplitudes at points indicated are listed from lowest to highest. The rise rate appears to be a direct function of concentration. The tail amplitude after the second rise is also a direct function of concentration, whereas the plateau amplitude becomes an inverse function at higher concentrations. This indicates that rise rate and plateau amplitude are distinct functions of concentration. (**D**) A quantitative analysis of the observations in C shows that the process underlying the plateau is specific to TA. The scatter-plot shows the amplitude of each individual response plotted against its maximum rise-rate. Data include all responses from experiments plotted in Figure 3C. The Spearman rank-order correlation (r_S_) between rise rate and peak amplitude for TA evoked responses is relatively weak (r_S_ = 0.52, p = 0.046), whereas for octopamine (OA) (r_S_ = 0.93, p = 2e–7) and dopamine (DA) (r_S_ = 0.97, p = 2e–7) it is strong. These correlations differ significantly between TA and the other two amines (TA vs OA, p = 0.002; TA vs DA, p = 0.0006) but not between OA and DA (OA vs DA, p = 0.382) (Fisher's z transformation for correlation coefficients, two-tailed Student's t-test). Each measurable response to an application of TA (4 oocytes, n = 15 responses), OA (6 oocytes, n = 24 responses), or DA (5 oocytes, n = 14 responses) was treated as an independent sample.

The limited amplitude of the tyramine response could not be attributed to a form of desensitization or down regulation requiring repetitive agonist application because the complex waveform including plateau appeared upon the first application of high concentration tyramine (Fig. 4, see also Fig. S1D). In addition, the plateau amplitude was not noticeably affected by repetitive tyramine application. Figure 4A shows a continuous recording where 100 µM tyramine is applied to an oocyte before proceeding with a concentration series that includes a second 100 µM application. Figure 4B shows the first 100 µM tyramine application (black) overlaid with the second 100 µM tyramine application (grey). The responses are aligned by the application of tyramine (black bar). Desensitization is evident in that the second rise and tail are of lower amplitude in the second response. Desensitization is also apparent in that the trace appears smoother because minor oscillations that occurred in the first (arrow) did not occur in the second. However, the delay of the response, the initial rise rate, and the amplitude of the plateau show almost no difference. Therefore, if the plateau is due to a desensitization mechanism, it must be a fast mechanism that occurs within seconds and is completely reversible within minutes.

The effect of the tyramine induced plateau on the maximal amplitude of I_D_ is illustrated further in Figure 4C. Currents evoked at concentrations from 0.1 µM to 1000 µM in 4A (color coded) are overlaid and aligned by the response onset in 4C. The peak amplitudes of responses above 10 µM are clearly limited by the appearance of the plateau even though the tail (after the second rise) of each response shows an increase in amplitude with concentration. The rate of the initial rise also increases as a direct function of tyramine concentration despite the correspondingly limited plateau amplitude. This indicates that the rise rate and plateau amplitude are distinct functions of concentration.

Figure 4D shows a quantitative description of this relationship. Rise rate is plotted against response amplitude for all I_D_ responses recorded as shown in Figures 3 and 4A. The scatter-plot shows that the peak amplitude of the tyramine response is limited despite an increasing rise rate. For tyramine-evoked I_D_ there is a significant but relatively poor Spearman correlation (r_s_) between rise rate and amplitude (r_S_ = 0.52, p = 0.046) (n = 15 responses from 4 oocytes). However for responses evoked by octopamine and dopamine the correlation is high and highly significant (OA: r_s_ = 0.93, p = 2e-7 [n = 24 responses from 6 oocytes]; DA: r_s_ = 0.97, p = 2e-7 [n = 14 responses from 5 oocytes]). Furthermore, these correlations differ significantly between tyramine and octopamine (p = 0.002), or tyramine and dopamine (p = 0.0006), but not between octopamine and dopamine (p = 0.382) (Fisher's z transformation for correlation coefficients, two-tailed Student's t-test). The correlations show that the maximum amplitude is a monotonic function of rise rate for octopamine and dopamine but not necessarily for tyramine.

From this we can conclude that a process separate from the initial rise limits the amplitude of tyramine-evoked I_D_, and is not significantly limiting to octopamine or dopamine-evoked I_D_ up to 1000 µM. This shows that the lower apparent efficacy of higher concentration tyramine in evoking I_D_ (Fig. 2B) is not because of a weakened response. It is due instead to the induction of an additional opposing process that underlies the plateau.

The observation of complex currents is not unusual for aminergic GPCRs expressed in oocytes (e.g. [46], [49]–[51]). However, to our current knowledge, it is a novel observation that a more complex waveform is evoked exclusively by tyramine and none of the other aminergic agonists. Our data cannot exclude the possibility that the additional complexity of the tyramine response is due to the action of an unidentified *Xenopus* GPCR. It is also possible that tyramine acts on some other unidentified endogenous protein such as an ion channel or transporter. However, this does not seem probable in light of the fact that I_D_ waveforms appear to be specific to the expressed receptor type. For example, in contrast to OA/TA_Mac_, octopamine was able to induce both oscillations and a reduced I_D_ at high concentration when the *Drosophila* OA/TA receptor (CG7485) was expressed in oocytes [51]. The response to octopamine in that case was similar to tyramine, hence the I_D_ showed no obvious indication of functional selectivity. If our observations are due to an endogenous receptor, as opposed to the activity of OA/TA_Mac_, it must be highly specific for tyramine, its expression must also be specifically induced according to the type of heterologously expressed receptor, and in addition it must not produce appreciable effects in uninjected oocytes.

### The pharmacological profile of OA/TA_Mac_ is similar to other octopamine/tyramine type receptors

**Figure 5:**
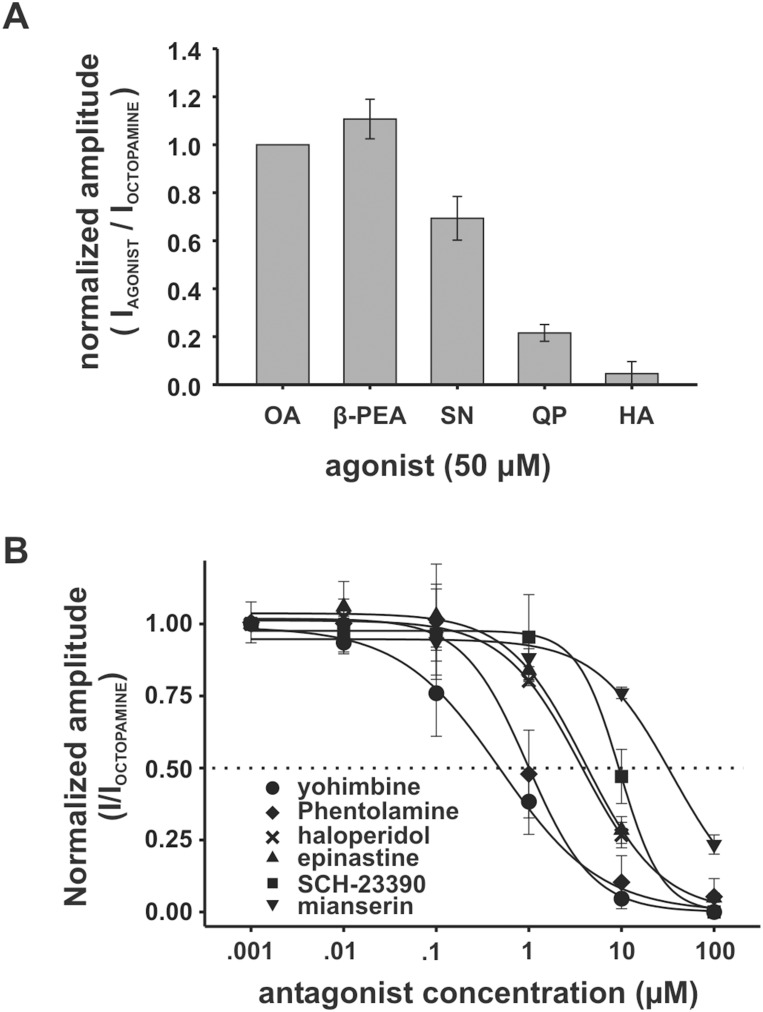
The pharmacological profile of OA/TA_Mac_. (**A**) The relative amplitude of agonist evoked direct-currents (I_D_) within single oocytes. (+/−)-octopamine (OA), β-phenylethylamine (β-PEA), (+/−)-synephrine (SA), (−)-quinpirole (QP), and histamine (HA) were all applied at 50 µM and normalized to the amplitude of the OA-evoked current. In this experimental set the response to OA ranged from 64 nA to 150 nA with a mean of 119 nA and a standard deviation of 32 nA (n = 6). (**B**) Relative efficacy of antagonists. Antagonists were applied as a mixture with 50 µM OA. The concentration series for each antagonist was tested on a different oocyte injected with OA/TA_Mac_ only and voltage clamped at −60 mV. Current amplitudes were normalized to the response at lowest antagonist concentration which was zero in most experiments. All antagonists were tested on 3 to 5 oocytes. Error bars in A and B represent the standard deviation of the normalized values.

To test agonists and antagonists associated with aminergic receptors we measured effects with respect to I_D_ evoked by a 30 second perfusion-switched application of 50 µM octopamine (Fig. 5). The octopamine response provided a more practical point of comparison than tyramine because of its larger amplitude (Fig. S1D) and lower variability at 50 µM. Also, as described above, tyramine induced effects not seen with any other agonist we tested. The set of agonists, all at 50 µM, were tested one after another in various orders on single oocytes (n = 6). At 50 µM the tyramine receptor agonist β-phenylethylamine (β-PEA) evoked a current of comparable or greater amplitude than octopamine (Fig. 5A). The dopaminergic agonist (−)-quinpirole and adrenergic agonist (+/−)-synephrine evoked smaller currents than octopamine or β-PEA. There was no response or minimal response evoked by histamine. Clonidine, an insect octopamine receptor agonist [52], was tested in prior experiments and was not included in this set because it also evoked no response (n = 4, Fig. S1C). We note that all included agonists were also tested individually in additional preliminary experiments (example traces shown in Fig. S1).

To test putative antagonists each was applied as a mixture with 50 µM octopamine using a 30 second perfusion-switched application. Of the antagonists tested, the adrenergic antagonist yohimbine was the most potent, reducing the octopamine response by half at a sub-micromolar concentration. Mianserin was the least potent, with an incomplete block at 100 µM (Fig. 5B). The relative rank of potency under these conditions was yohimbine (IC_50_≈0.48 µM) > phentolamine (IC_50_≈0.98 µM) > epinastine (IC_50_≈3.70 µM)≈haloperidol (IC_50_≈4.20 µM) > SCH-23390 (IC_50_≈9.60 µM) > mianserin (IC_50_≈32.0 µM). Representative current traces from these experiments are shown in Figure S5.

The sensitivity to tyramine, octopamine and dopamine (Fig. 3), and the pharmacological profile, are similar to that of other characterized OA/TA tyraminergic (TyrR1) type receptors [33], [35], [43], [51]. Both tyramine and the tyramine receptor agonist β-PEA is the most effective in evoking I_D_ and yohimbine is the most potent antagonist of octopamine evoked I_D_. We also note that both the *Bombyx mori* [33] and *Drosphila* [51] OA/TA (TryR1) receptors are also responsive to dopamine while the *Drosphila* [24] and *Bombyx* [25] TryR2 are not. The α-adrenergic-like OA1 octopamine receptors are also non-responsive to dopamine [27], [45]. These results establish that the functional attributes so far observed for OA/TA_Mac_ are consistent with its previously reported phylogenetic grouping as an OA/TA (or TyrR1) type receptor [14].

### Differential modulation of the transient chloride current I_Cl-T_ by tyramine and octopamine

**Figure 6:**
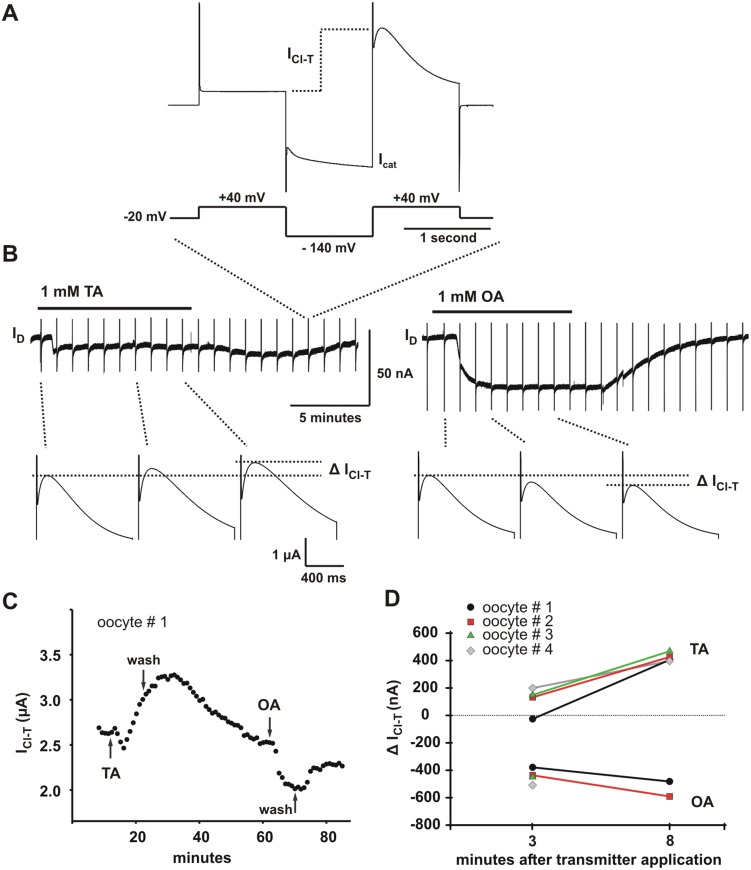
Differential modulation of a transient chloride current by tyramine (TA) and octopamine (OA). I_Cl-T_ is an endogenous calcium-dependent transient chloride current coupled to extra-celluar calcium influx. It is thought to arise from a single type of channel [53]. At a high concentration tyramine (TA) reversibly increases I_Cl-T_ and octopamine (OA) reversibly decreases it. (**A**) The voltage command protocol used to measure I_Cl-T_ is shown with an example current. The first step to +40 mV from the holding potential of −20 mV evokes no I_Cl-T_ and provides the baseline. The hyperpolarizing step to −140 mV induces calcium influx that causes I_Cl-T_ to appear during the second step to +40 mV [53]. The amplitude of I_Cl-T_ was measured as the difference between the first step and the peak of the transient current evoked by the second. (**B**) Measurements of I_Cl-T_ were taken every minute during the I_D_ evoked by long applications of 1 mM TA and 1 mM OA. The vertical lines are the currents evoked by the voltage command pulse used to measure I_Cl-T_ (as shown in A). Their true amplitude was clipped from the image during figure preparation. Below the trace individual I_Cl-T_ transients are shown. Δ I_Cl-T_ is the difference in amplitude of these transients measured at different time points. The amplitude of I_Cl-T_ increases during continuous TA application and decreases during continuous OA application. There was a 38 minute washout period between the TA and OA applications. (**C**) All measurements of I_Cl-T_ taken over the entire duration of the experiment shown in B. (**D**) Data from 4 different oocytes showing Δ I_Cl-T_ at the 3 and 8 minute post-transmitter application time points. Data from oocyte #1 are shown in B and C. The 8 minute time point was not taken for the OA response of oocytes #3 (Fig. S2) and #4.

The fluctuations in holding current that we refer to as I_D_ have often been referred to as the calcium-dependent chloride current. These currents are in fact complex and are known to contain multiple poorly defined components that arise from a number of unidentified potassium, chloride and mixed cationic channels [46], [47]. For this reason, we tested the effect of tyramine and octopamine on one of the better characterized components, the transient calcium-dependent chloride current that we refer to as I_Cl-T_ after [53]. This current, which has been referred to as I_out_ [50], [54], I_Cl-2_ [55] or I_Cl1-T_ [53], can be clearly isolated from I_D_ using a specific pulse protocol (Fig. 6A) [53]. Furthermore, I_Cl-T_ is known to increase in response to inositol 1,4,5-triphosphate (IP_3_) injection [53], [55]. Modulation of this current therefore provides additional insight into how IP_3_ related pathways might be affected by transmitter application.

I_Cl-T_ was measured under two-electrode voltage-clamp by hyperpolarizing the oocyte to −140 mV after a step to +40 mV and stepping back to +40 mV (Fig. 6A). The size of the transient current observed during the second step to +40 mV depends on the amount of calcium entry that occurred through the calcium permeable cation conductance (I_cat_) during the hyperpolarizing step to −140 mV [47], [54]. A change in the size of I_Cl-T_ thereby gives a measure of channel modulation. I_Cl-T_ is measured instead of I_cat_ because I_Cl-T_ is a pure chloride conductance that likely arises from one ion channel population while I_cat_ is complex arising from more than one conductance [54].

We monitored I_Cl-T_ over the course of extended transmitter applications using the described pulse protocol. During prolonged applications of 1 mM tyramine I_Cl-T_ increased, whereas during prolonged applications of 1 mM octopamine it decreased (n = 4) (Fig. 6 B–D and Fig. S2). Figure 6B illustrates this effect in detail by showing a close up of the I_D_ response along with expanded current traces of I_Cl-T_ taken at the indicated time points. All of the measurements of I_Cl-T_ taken over the entire course of the recording are plotted in Figure 6C. Figure 6D shows the results from 4 different oocytes that were subjected to similar applications. The change between the last pre-application measurement and subsequent time points (Δ I_Cl-T_), at 3 and 8 minutes post-transmitter application, are shown for each individual oocyte. In all cases tyramine caused an increase in I_Cl-T_ while octopamine caused a decrease. These four recordings were done subsequent to the initial observation of this effect in other OA/TA_Mac_ receptor-injected oocytes and the effect was absent in uninjected oocytes.

The effect of both tyramine and octopamine on I_Cl-T_ was also tested at 1 µM and 100 µM (Fig. S3; n = 5 at each concentration). Since tyramine produces distinctly different effects on I_D_ at low (below 10 µM) and high concentrations we might expect the same for I_Cl-T_, however, this was not the case. Tyramine also produced an increase in I_Cl-T_ at lower concentration, while octopamine produced no apparent effect. In addition, the strength of the tyramine-induced increase appeared to be dose dependent (Fig. S3 C). The fact that tyramine evokes increases in I_Cl-T_ independent of concentration suggests that the differential effect at 1 mM is unlikely to result from the increase in fractional activation of the receptor population. Furthermore, the relationship between the I_D_ plateau and I_Cl-T_, if any, is unclear.

These experiments demonstrate clear differential modulation of I_Cl-T_, a current that has a well described calcium dependence [47], [53], [55]–[57]. However, we emphasize that increases and decreases in I_Cl-T_ cannot be interpreted to directly indicate rising and falling calcium levels due to intracellular release. I_Cl-T_ is specifically coupled to the influx of extracellular calcium that occurs through the mixed cation current (I_cat_) [47], [54]. I_cat_ can be modulated in parallel with intracellular calcium release [53]. Thus, the modulation we observe is expected to coincide with calcium modulation but does not necessarily depend on it. While not strictly indicative of intracellular calcium levels, these experiments do provide strong evidence that tyramine, at high concentration, stimulates a PLC/IP_3_ linked pathway through OA/TA_Mac_ that octopamine does not.

The opposite action of tyramine and octopamine on I_Cl-T_ also provides evidence that antagonistic modulation of the underlying pathways may minimally require a single receptor type. This finding merits further investigation in an *in vivo* cellular environment where endogenous OA/TA (TryR1) receptors are expressed.

### What are the cellular and/or molecular mechanisms that give rise to the observed functional selectivity?

Functional selectivity is indicated through the measurement of I_D_ by the fact that tyramine evokes an additional process at high-concentration that octopamine does not. As shown in Figure 4, this can be inferred solely from the characteristics of the I_D_ waveforms. Functional selectivity is also indicated in the simultaneous measurement of I_D_ and I_Cl-T_ shown in Figure 6B and S7. At high concentrations octopamine appears to be more effective in increasing I_D_ while tyramine is more effective in increasing I_Cl-T_. Furthermore, both octopamine and tyramine increase I_D_ while they have opposite effects on I_Cl-T_ (Fig. 6B). Thus, a simple two-state model of receptor activation does not appear to be compatible with either I_D_ or I_Cl-T_. Figure 6B provides a particularly strong demonstration of functional selectivity because both effects are measured simultaneously and continuously for both ligands within single cells.

The simplest explanation of the observed functional selectivity is that tyramine enables at least one additional receptor state that octopamine does not. We note that multiple active receptors states cannot be absolutely proven without more direct structural or binding data. Alternative explanations include the presence of an unknown receptor as discussed above or more complex downstream effects of intracellular signaling components. Based on our observations, the process underlying the I_D_ plateau does not appear to be a downstream effect of either calcium or cAMP.

It is well established that induction of endogenous currents in *Xenopus* oocytes indicates potential coupling to Gα_q_/phospholipase C (PLC) mediated pathways [58]. In addition, as discussed above, I_Cl-T_ is activated by IP_3_ injection [53]. The probable involvement of these PLC related signaling components suggests we should expect changes in intracellular calcium levels to occur with OA/TA_Mac_ activity. The current oscillations that appear to be specifically triggered by tyramine (Figures 3 and 4) also suggest effects on intracellular calcium levels. After extended incubation with thapsigargin in calcium-free medium, a procedure known to purge calcium from the endoplasmic reticulum [59], oscillations were not observed (Fig. S4). However, the underlying tyramine-induced I_D_ waveform (plateau and second rise) appeared to be unaffected. Thus we have no evidence that the functional selectivity we observe is a secondary effect of intracellular calcium release.

It is also unlikely that the effects of tyramine at high concentration result from an increase in cAMP. It is common that octopaminergic GPCRs can couple through the canonical Gα_s_/cAMP pathway so we used co-expression of the Cystic Fibrosis Trans-membrane Conductance Regulator (CFTR) as a means of testing for increases in cAMP (see methods for more detail) [60]–[63]. Our measurements indicated that neither octopamine nor tyramine produced significant increases in cAMP levels (Fig. S5). Furthermore, the application of the adenylate cyclase inhibitor SQ-22536 had no apparent effect on I_D_ (tyramine [n = 3], octopamine [n = 6]) (Fig. S6).

While functional selectivity is clearly indicated by differential effects of tyramine and octopamine on both I_D_ and I_Cl-T_, elucidation of the underlying mechanisms remains a non-trivial matter. Future work towards better defining the underlying mechanisms may enable the relatively rapid assessment of a receptors tendency towards biased signaling using electrophysiology in the oocyte system.

### Remarks on the implications for *in vivo* receptor function

Despite growing evidence that functional selectivity (or biased agonism) and concentration-sensitive functional selectivity are conserved features of arthropod aminergic receptors, the importance of these properties for nervous function remains to be determined. An important physiological implication of concentration-sensitivity is that it can enable the location of a receptor to determine its intracellular effect. Receptors located at or near the synaptic cleft can be exposed to transmitter concentrations in the millimolar range [64]. Receptors located far from release sites are exposed to lower concentrations. In crustaceans this principle is well established for dopamine based on morphological data (See for example [65], [66]). In addition, octopamine is well understood to function in a hormonal capacity in crustaceans [11], [67], [68]. While growing evidence indicates that tyraminergic neurotransmission occurs in arthropods [17], [18], [69]–[72], specific synaptic and hormonal functions remain obscure. Our findings raise the possibility that single-type OA/TA receptors can produce different cellular effects in each capacity.

The extent to which OA/TA type receptors may be modulated via exposure to both tyramine and octopamine *in vivo* is also unclear. Mixed release seems likely to occur in at least some octopaminergic neurons because tyramine is a precursor of octopamine. The possibility of differential effects of tyramine and octopamine being mediated through a single receptor was first proposed based on biased agonism observed for the *Drosophila* OA/TA type receptor [43]. Our results also suggest that cellular effects at high concentration (such as at the synapse) will be variable as a function of the patterning of the exposure. Because tyramine evokes an additional opposing process we can predict a nonlinear summation of effect. This means that the wave-form of a post-synaptic potential could in principle be modulated by the relative timing or ratio of tyramine and octopamine (see S9).

It is of primary concern to determine if differential effects – and as a result emergent behaviors – may normally occur *in vivo* through the single receptor type. While some effects of tyramine have been observed to oppose those of octopamine in honeybee and *Drosophila* [17], [19], [20], [22], the behavioral effects of tyramine on agonistic encounters in prawn have yet to be determined. The OA/TA_Mac_ receptor is likely important in nervous function because it is present throughout the prawn's nervous system [14] as are neurons containing octopamine [73]. However, a specific synapse or cell where *in vivo* tyramine signaling can be studied in prawn remains to be identified.

## Materials and Methods

### Molecular constructs and RNA synthesis

The full length coding region of the OA/TA_Mac_ receptor (EU233816) was prepared for subcloning by PCR amplification. The primer 5′-GA TGA TCA GAA GAA ATG ACC CGC TTT AAG CTT CTC-3′ was used to add a BclI restriction site and a Kozak region to the 5′-end; and the primer 5′-GA TGATCA CTA CAC TGT TGC AGC ATT-3′ was used to add the BclI restriction site to the 3′-end. OA/TA_Mac_ was subcloned into pBSTA plasmid [74] which was then used as template for RNA synthesis. Capped cRNA was synthesized using the mSCRIPT kit (Epicenter, Madison, WI). Integrity of the RNA was checked on an agarose gel and concentration was determined by absorbance using a NanoDrop ND 1000 spectrophotometer. RNA was adjusted to 1.0 µg/µL in water and stored in aliquots at −80°C. The cystic fibrosis transmembrane conductance regulator (CFTR, NM_000492) was prepared as for OA/TA_Mac_.

### Oocyte injection

Mature stage V or VI *Xenopus laevis* oocytes were collected, following procedures in strict accordance with the recommendations in the Guide for the Care and Use of Laboratory Animals of the National Institutes of Health, as described and approved by the University of Puerto Rico Medical Sciences Campus Institutional Animal Care and Use Committee (IACUC) in protocol 3240104. All surgery to collect oocytes was performed under tricaine anesthesia, and all efforts were made to minimize pain and suffering. The collected oocytes were maintained in ND 96 (96 mM NaCl, 2 mM KCl, 1.8 mM CaCl_2_, 1 mM MgCl_2_, 5 mM HEPES, pH 7.6) supplemented with antibiotics (Tetracycline 50 mg/L and Amikasin 330 mg/L) at 18°C. The follicle was removed mechanically following a collagenase treatment before injection with 50 nL of poly-A cRNA. OA/TA_Mac_ was injected at concentrations up to 0.15 µg/µL. In other experiments, a mixture of 0.1 µg/µL OA/TA_Mac_ and 0.025 µg/µL CFTR was used or up to 0.05 µg/µL CFTR alone was used.

### Electrophysiology

Recordings were performed 1–3 days post-injection at room temperature (20–22°C). Currents were recorded under two-electrode voltage clamp using an Axoclamp 900a amplifier (Molecular Devices LLC, CA USA). A digidata 1440a analog to digital converter was used in conjunction with pCLAMP 10 software (Molecular Devices) for data acquisition and to generate voltage commands. For each step protocol or chemical application data were typically sampled at 5 KHz and filtered digitally at 1 KHz. For the duration of each experiment the current was also continuously sampled at 1 kHz using a separate minidigi 1A digitizer (Molecular Devices). Glass microelectrodes were pulled from filamented 1.2 mm thin wall borosilicate glass (World Precision Instruments, Sarasota, FL) using a Sutter Instruments P-97 puller. They were filled with 3 M KCl and had resistances of 0.8–2.0 MΩ. A silver chloride pellet immersed in 3 M KCl and connected to the bath via a 3% agar bridge made with 3 M KCl was used as the reference electrode.

The experiments shown in Figure S7 were done separately by EcoCyte Bioscience, Houston, TX. We provided the experimental design and OA/TA_Mac_ cRNA. EcoCyte performed the experiments and returned the raw trace data for analysis. *Xenopus laevis* oocytes were prepared by standard methods similar to those described above. RNA injections and recordings were done using the Roboocyte multichannel recording and injection system (Multi Channel Systems, Reutlingen Germany). Recordings were done 2–3 days post-injection at room temperature in standard frog saline (90 mM NaCl, 2 mM KCl, 2 mM CaCl_2_, 1 mM MgCl_2_, 5 mM HEPES, pH 7.6).

### Test compounds

Compounds were obtained from Sigma and dissolved in ND96 or calcium free ND 96 (96 mM NaCl, 2 mM KCl, 5 mM MgCl_2_, 0.1 mM EGTA, and 5 mM HEPES, pH 7.6), unless stated otherwise. Water-soluble compounds, including (+/−)-octopamine hydrochloride, dopamine hydrochloride, and tyramine hydrochloride, were made fresh as 1 M stocks the day of the experiment and serially diluted. Epinastine was prepared as a 0.06 M stock in water and stored at −20°C. SQ-22536 was prepared as a 0.1 M stock in water. Yohimbine was dissolved in acidic ND 96 to 1 mM, before being serially diluted at pH 7.6. Forskolin was prepared as a 0.05 M stock in DMSO. Thapsigargin was prepared as a 0.01 M stock in DMSO. (−)-Quinpirol was made as a 0.01 M stock in ND96 and stored in aliquots at −20°C. Haloperidol, on the day of the experiment, was dissolved in methanol at 70°C to a concentration of 0.01 M. The methanol solution was then diluted 1000 fold in ND 96 at 70°C with vigorous vortexing to yield a 10 µM solution from which serial dilutions in ND 96 at room temperature were made. Haloperidol could not be tested at 100 µM because of its limit of solubility. For all antagonists serial dilutions were made in ND 96 containing 50 µM octopamine and applied as a mixture.

### cAMP detection and the determination of whole oocyte conductance

As a means of detecting rises in intracellular cAMP levels we tested oocytes co-expressing the CFTR channel and OA/TA_Mac_. Heterologously expressed CFTR can be used as an extremely sensitive assay for intracellular cAMP in *Xenopus* oocytes [62]. CFTR mediates a chloride-selective leak conductance that is activated by increases in intracellular cAMP [63]. Activity of CFTR can thus be used to indicate whether changes in cAMP correspond with ligand application [60]–[62]. Whole oocyte conductance was monitored using a step protocol and calculated from I/V plots. The command voltage was stepped from the holding potential to various levels (−10 mV to −55 mV in −5 mV increments) for 150 ms. The steady state current during each step was plotted against the command voltage and fit to a line using Clampfit 10 software (Fig. S7A). The slope of the line was taken as the conductance. To minimize the magnitude of the holding current between drug applications, oocytes expressing CFTR were typically held at −20 mV which is close to the typical chloride reversal potential. This was confirmed by the fact that the direct-current evoked by forskolin could appear inward or outward at −20 mV. Oocytes expressing either OA/TA_Mac_ alone or OA/TA_Mac_ and CFTR typically had resting membrane potentials between −20 mV and −30 mV (mean of −26±5 mV calculated from oocytes in Fig. S7) due to increased expression of endogenous chloride currents [47].

### Perfusion of test compounds

Oocytes were continuously perfused at 1.5 mL/min either with ND 96 or test compounds dissolved in ND 96. Two types of application were used over the course of this study, a perfusion-switch or a focal application. In all cases solutions were delivered through a gravity fed system and switched using a computer controlled bank of pinch valves. Flow rate was regulated with a manual screw-type pinch valve.

For focal application a triple barrel pipette with three parallel independently switched channels was positioned about 1 mm from the surface of the oocyte, up-stream of a cross flow, at an approximate angle of 45 degrees (schematic shown in Fig. S8). Drugs were focally applied during continuous perfusion with ND 96. This exposed the oocyte to a rapid pulse that immediately and almost completely enveloped the oocyte, as determined by observing the application of dye colored solution. Thus the focal application produced an exposure at the applied concentration, over most of the oocyte surface, for a duration equaling the approximate duration of the application. Focal applications produced vigorous responses with a shorter delay and longer washout time than perfusion-switched applications.

In experiments where more than three drugs or concentrations were tested a precisely controlled 30 s perfusion-switch was used. Up to eight channels were connected to the bath via an 8-way manifold. There was an approximately 6–7 s delay for solutions to traverse the dead volume between the manifold and the bath. During a switch, to prevent back-flow, the open channel was turned off at least 100 ms before the next was turned on. To determine the concentration profile for this type of application we monitored the resistance of a bath electrode while flowing deionized water, and then switching to 3 M NaCl for 30 s. By observing the drop in resistance relative to that in pure 3 M NaCl we determined that the maximum concentration was reached at approximately 30 seconds. This resulted in a concentration profile where the test compound flowed through the bath for approximately 1 minute but only momentarily reached the applied (peak) concentration near 30 s. For concentration series a 5–8 min washout was used in between perfusion-switched applications.

### Data analysis and figure preparation

Analysis of trace data was done using pCLAMP 10 software (Molecular Devices). Data plots and curve fits were done using pCLAMP 10 or SigmaPlot 11 software. EC_50_ and IC_50_ values are estimates obtained graphically. Final figures were prepared using CorelDRAW. Images of electrophysiological traces and graphs were imported into CorelDRAW from the analysis programs.

Statistical tests and calculations of Spearman correlations were done using Sigmaplot 11 software. Not all data were normally distributed as determined by the Shapiro-Wilk test. For this reason non-parametric tests were performed as stated in the figure legends. Within oocyte comparisons in the designed experiment of Figure S2 were treated as paired values. Compiled data from different preliminary experiments shown in Figure S3 were treated as independent samples. Significance testing on the difference between correlation coefficients was done using Fisher's z-transformation method for correlation coefficients [75] followed by the two-tailed t-test as implemented in the cocor: Comparing Correlations R-package [76].

## Supporting Information

Figure S1 **Direct-current (I_D_) responses are specifically evoked by agonists in oocytes injected with OA/TA_Mac_ only.** (**A**) Un-injected oocytes do not respond to dopamine (DA), octopamine (OA), or tyramine (TA). The trace is representative of an experimental set of 5 oocytes. Additional uninjected oocytes were tested with various experimental sets throughout the course of this study. (**B**) A typical preliminary test of agonist sensitivity. Injection of OA/TA_Mac_ cRNA alone is sufficient to confer sensitivity to OA. The putative agonist quinpirole (QP) also produces a response in injected oocytes and not in uninjected oocytes. Applications are approximately 30 seconds in the upper trace. (**C**) The response of clonidine (CN), synephrine (SN), and OA within a single OA/TA_Mac_ injected oocyte. Clonidine was tested on 4 injected oocytes and produced no visible response. (**D**) A representative current trace from the experimental set shown in Fig. 5A. All drugs are applied for 30 seconds each as indicated by black rectangles. Agonist evoked currents are measured within oocytes relative to the octopamine response at 50 µM. The comparison is made to OA because the amplitude of the tyramine response becomes small at concentrations above 10 µM. This is due to a mechanistically undefined process not seen with any other agonist we tested. Note that TA is the first compound applied to a naïve oocyte in this example. Histamine (HA) also produced a minimal response similar to CN in preliminary experiments.
(TIF)

Figure S2 **The full detail for the experiment of oocyte #3 in** **Figure 6D** **showing the effect of tyramine (TA) and octopamine (OA) on I_Cl-T_.** (**A**) The entire recording shown at full scale. The oocyte is voltage clamped at −20 mV. (**B**) The I_D_ response for both TA and OA. Vertical lines are the simultaneously measured I_Cl-T_. The net conductance change for the TA response is typically near zero (see. Fig. S5 C1) causing the I_D_ to be small, especially at the holding potential of −20 mV, which is near the chloride reversal potential. In this example the TA-evoked I_D_ is in the range of baseline fluctuations in holding current. (**C**) An overlay of the first 10 recordings of I_Cl-T_ during tyramine application (TA). There is minimal change in the amplitude of the first step which is the reference for calculating peak height of the second step. I_cat_ is a mixed cation current that is mediated by multiple unidentified ion channels. The I_Cl-T_ transient is probably mediated by a single channel type and is dependent on both voltage and influx of extracellular calcium through I_cat._ (**D**) I_Cl-T_ shown for all measurements in A. Note that I_Cl-T_ continues to increase or decrease long after the I_D_ responses reach their respective plateaus. In other words I_D_ saturates before I_Cl-T_. The difference in amplitude of I_D_, between TA and OA (B), is well within 100 nA, while the difference in I_Cl-T_ is over 1000 nA (D). This is a clear indication that the time course and maximum amplitude of I_D_, as discussed per Fig. 2, cannot faithfully reflect fractional ligand binding or receptor ‘activation’.
(TIF)

Figure S3 **The effect of tyramine (TA) and octopamine (OA) on I_Cl-T_ at 1 µM and 100 µM.** (**A1** and **A2**) Recordings of I_D_ from two different oocytes showing 8 minute applications of biogenic amines (black bars). The oocytes are voltage clamped at −20 mV and a measurement of I_Cl-T_ was taken every minute. The pulses used to measure I_Cl-T_ appear as vertical lines and are numbered. (**B1** and **B2**) Individual measurements of I_Cl-T_ from the corresponding traces in A1 and A2. (**C**) The mean responses from 5 different oocytes at each concentration. Values for each pulse are normalized to the smallest amplitude pulse during the first 8 minutes. Error bars represent the standard deviation of the normalized values and are in one direction for 100 µM responses for clarity.
(TIF)

Figure S4 **Pre-incubation in thapsigargin in calcium-free solution had no apparent effect on the tyramine induced plateau.** The oscillations that were sometimes seen in normal saline (ND96) were not seen in calcium free saline. However, no obvious effects on the underlying I_D_ waveform were seen when experiments were done in calcium-free ND96, indicating that extracellular calcium influx is not required for the development of the plateau. To specifically deplete calcium from endoplasmic reticular stores, oocytes were incubated in 1.7 µM thapsigargin in calcium-free ND 96 for three hours [59]. Under these conditions the response to tyramine was still observed and was of a similar waveform in that a plateau and second rise were still apparent (n = 2). Vertical lines are I/V pulse protocols used to monitor conductance. Measured values are given below.
(TIF)

Figure S5 **Biogenic amines do not evoke significant changes in CFTR conductance (g).** (**A**) The measurement of whole oocyte conductance was done by plotting steady state current against the command voltage recorded during a step protocol. It is defined as the slope (I/V) of the linear least squares regression line. In the example shown tyramine (TA) causes no apparent change in whole oocyte conductance. Octopamine (OA) causes a small change compared to the adenylate cyclase activator forskolin (FSK). (**B**) TA or OA evoke comparable changes in conductance in oocytes injected with either OA/TA_Mac_ only, or OA/TA_Mac_ and CFTR (not significant [n.s.], p = 0.437, Mann-Whitney Rank-Sum Test). FSK evokes a significantly larger conductance than baseline or TA (**p = 0.0003) but not OA ([n.s.] p = 0.066, Mann-Whitney Rank-Sum Test). These cumulative data are from 20 OA/TA_Mac_ and CFTR-injected oocytes and 6 OA/TA_Mac_ -only injected oocytes. Conductance was measured as shown in A. Not all compounds were tested in all oocytes. Each response is treated as an independent sample. Concentrations of OA and TA were at 100 µM or 1000 µM, FSK was at 50 µM. Some points obtained at 3 minutes were grouped with the 4 minute responses. (**C**) The change in conductance (Δg) occurring within oocytes as determined by the difference between baseline and the indicated time points post-application of compound. The mean conductance for TA responses compiled from 12 oocytes was 0.30 µS, s.d. = 1.72 µS (high conductance outlier excluded). These values were significantly different between all responses (**TA vs OA, p = 0.002; ***TA vs FSK, p = 0.001; *OA vs FSK, p = 0.045) (Mann-Whitney Rank-Sum Test). (**D**) A subset of data from C showing 4 oocytes on which all three compounds were tested. The within oocyte comparison is the most appropriate and informative because it minimizes the experimental error.
(TIF)

Figure S6 **The adenylate cyclase blocker SQ-22536 produces no obvious effect on amine evoked direct-current (I_D_) or conductance.** (**A**) Tyramine (TA) (n = 3). (**B**) Octopamine (OA) (n = 6). Vertical lines are I/V pulse protocols used to monitor conductance. Measured values are given below each vertical line.
(TIF)

Figure S7 **Example current traces from experiments testing putative antagonists and used to generate** **Figure 5B** **.** Amplitudes plotted in Fig. 5B were normalized to the amplitudes at lowest concentration. Antagonists were applied as a mixture with 50 µM octopamine. All applications were for 30 seconds.
(TIF)

Figure S8 **At 10 µM tyramine (TA) is more effective at evoking a direct-current (I_D_) response than octopamine (OA) or dopamine (DA) within single oocytes.** Transmitters were applied in various orders using a 10 second focal applications indicated by arrow heads (schematic at top). The TA response was variable between oocytes (229±152 nA) and ranged from 96 nA to 563 nA. The OA (75±55 nA) and DA (46±31 nA) responses were correspondingly variable (mean ± s.d.) (n = 11 oocytes). (**A**) Despite between cell variability the TA response was invariably the largest within single oocytes. Representative current traces from three different oocytes are shown. (**B**) The mean response amplitude recorded as in A (***TA vs [OA or DA], p = <0.0001; *DA vs OA, p = 0.042). (**C**) The mean rise rate from the same responses shown in A and B (**TA vs OA, p = 0.002; ***TA vs DA, p = <0.001; *DA vs OA, p = 0.032). The rise rate was calculated by fitting a line to the linear portion of the rise. Data in B and C were treated as paired comparisons within single oocytes using Wilcoxon's signed-rank test. Error bars in B and C represent standard error of the mean. Oocytes were co-injected with OA/TA_Mac_ receptor and CFTR cRNA and voltage clamped at −60 mV.
(TIF)

Figure S9 **Dual exposure to octopamine (OA) and tyramine (TA) can produce variable wave-forms based on arbitrary pattering.** The ratio and timing of OA and TA can modulate the membrane potential across a range. (**A**) At high concentration TA can induce an opposite and dominant effect to OA on membrane potential. During a long application of 100 µM OA the membrane potential appears to reach a stable level. After switching to an equally concentrated mixture of 100 µM OA and 100 µM TA the potential is reduced below baseline. (**B1**) A 5 second pulse of TA alone results in a bi-phasic response with a low amplitude peak. (**B2**) A 5 second pulse of OA alone produces a larger amplitude mono-phasic response that is clearly different. (**B3**) Arbitrary patterning of these applications can produce other wave-forms.
(TIF)
