# Supplementary figures and images for: Characterization of a Prawn OA/TA Receptor in Xenopus Oocytes Suggests Functional Selectivity between Octopamine and Tyramine
Source: PLoS One. 2014 Oct 28;9(10):e111314. doi: 10.1371/journal.pone.0111314 (PMC4211885; doi:10.1371/journal.pone.0111314)

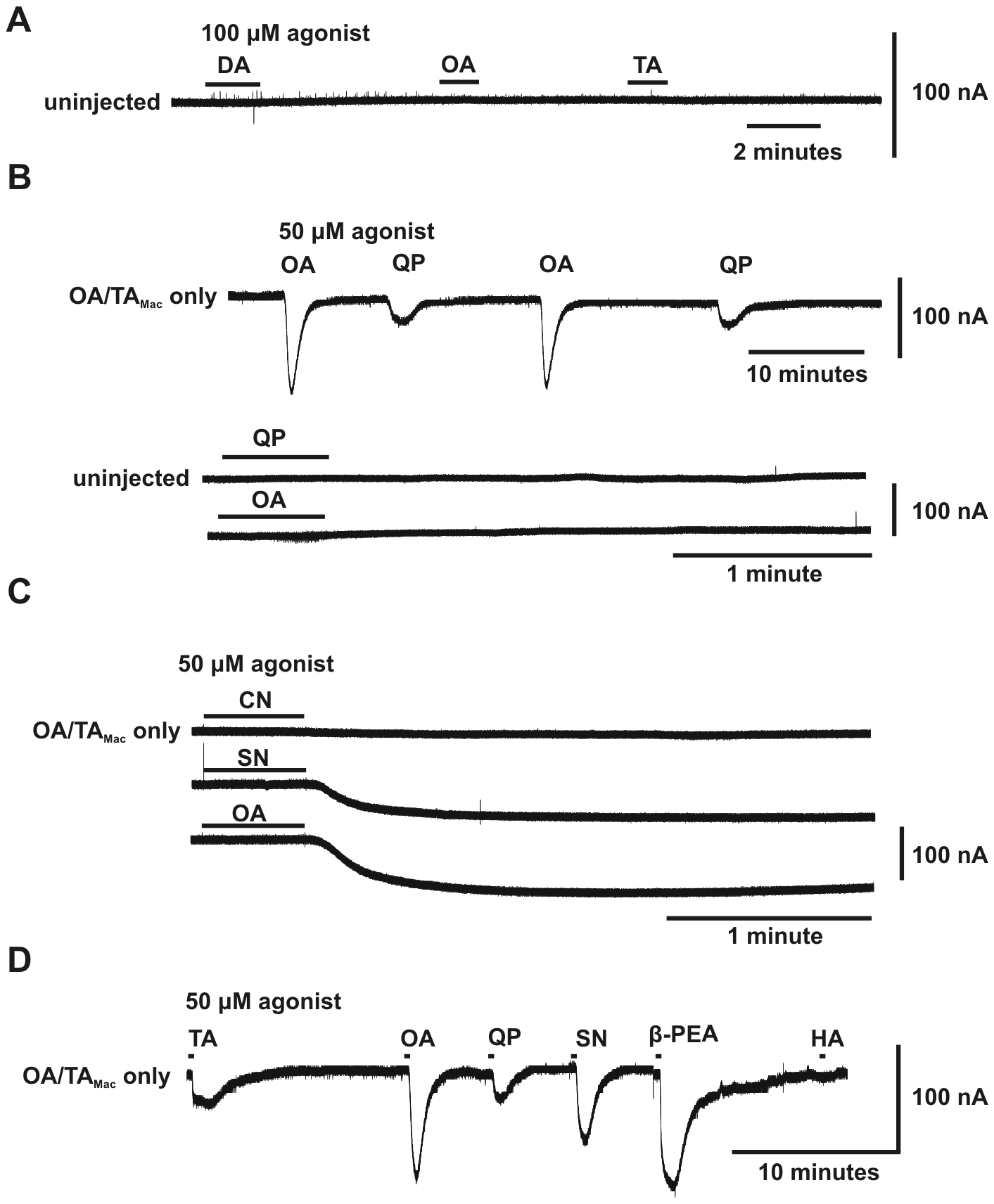

Supplement: Figure S1 — Direct-current (ID) responses are specifically evoked by agonists in oocytes injected with OA/TAMac only. (A) Un-injected oocytes do not respond to dopamine (DA), octopamine (OA), or tyramine (TA). The trace is representative of an experimental set of 5 oocytes. Additional uninjected oocytes were tested with various experimental sets throughout the course of this study. (B) A typical preliminary test of agonist sensitivity. Injection of OA/TAMac cRNA alone is sufficient to confer sensitivity to OA. The putative agonist quinpirole (QP) also produces a response in injected oocytes and not in uninjected oocytes. Applications are approximately 30 seconds in the upper trace. (C) The response of clonidine (CN), synephrine (SN), and OA within a single OA/TAMac injected oocyte. Clonidine was tested on 4 injected oocytes and produced no visible response. (D) A representative current trace from the experimental set shown in Fig. 5A. All drugs are applied for 30 seconds each as indicated by black rectangles. Agonist evoked currents are measured within oocytes relative to the octopamine response at 50 µM. The comparison is made to OA because the amplitude of the tyramine response becomes small at concentrations above 10 µM. This is due to a mechanistically undefined process not seen with any other agonist we tested. Note that TA is the first compound applied to a naïve oocyte in this example. Histamine (HA) also produced a minimal response similar to CN in preliminary experiments. (TIF) [file pone.0111314.s001.tif]

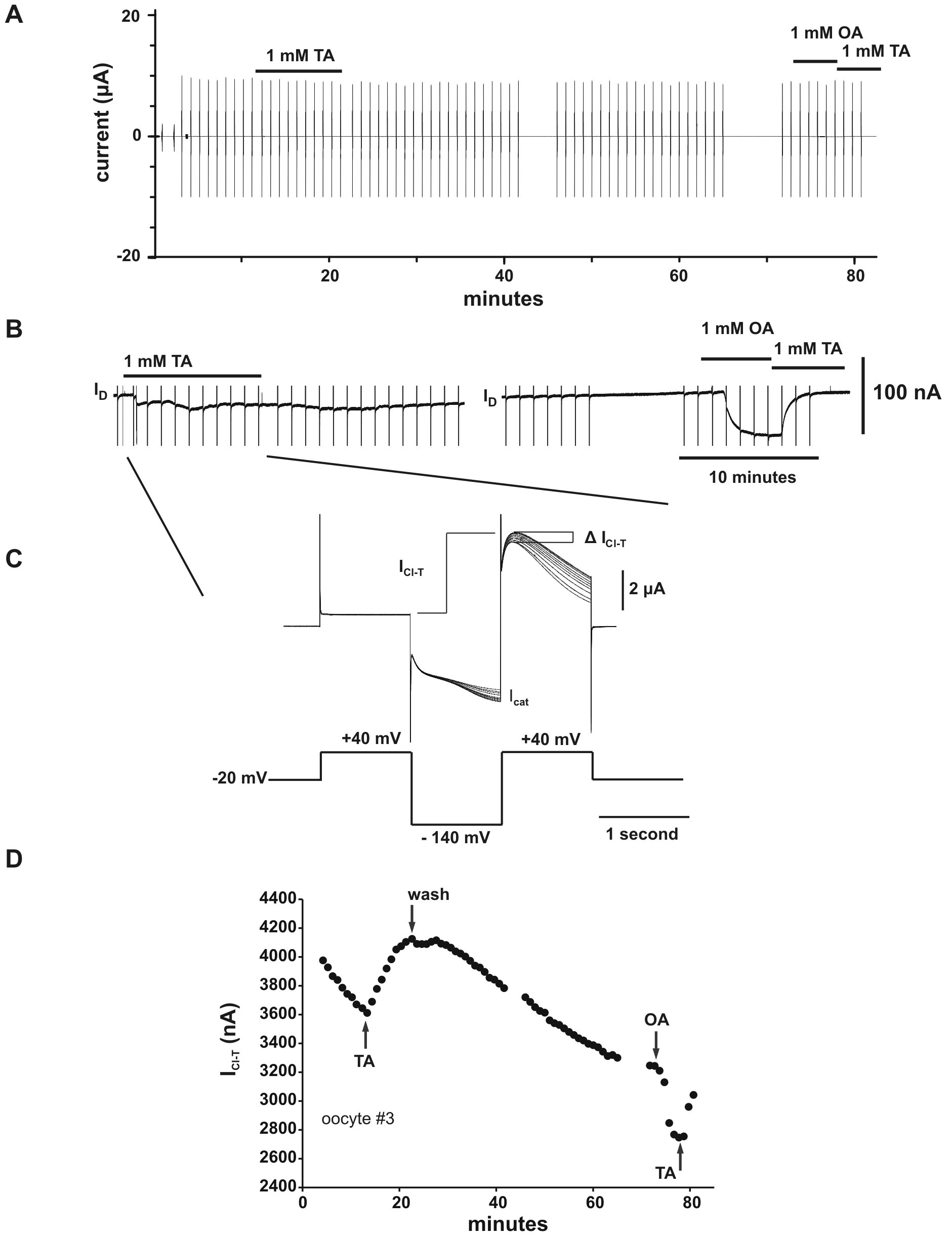

Supplement: Figure S2 — The full detail for the experiment of oocyte #3 in Figure 6D showing the effect of tyramine (TA) and octopamine (OA) on ICl-T. (A) The entire recording shown at full scale. The oocyte is voltage clamped at −20 mV. (B) The ID response for both TA and OA. Vertical lines are the simultaneously measured ICl-T. The net conductance change for the TA response is typically near zero (see. Fig. S5 C1) causing the ID to be small, especially at the holding potential of −20 mV, which is near the chloride reversal potential. In this example the TA-evoked ID is in the range of baseline fluctuations in holding current. (C) An overlay of the first 10 recordings of ICl-T during tyramine application (TA). There is minimal change in the amplitude of the first step which is the reference for calculating peak height of the second step. Icat is a mixed cation current that is mediated by multiple unidentified ion channels. The ICl-T transient is probably mediated by a single channel type and is dependent on both voltage and influx of extracellular calcium through Icat. (D) ICl-T shown for all measurements in A. Note that ICl-T continues to increase or decrease long after the ID responses reach their respective plateaus. In other words ID saturates before ICl-T. The difference in amplitude of ID, between TA and OA (B), is well within 100 nA, while the difference in ICl-T is over 1000 nA (D). This is a clear indication that the time course and maximum amplitude of ID, as discussed per Fig. 2, cannot faithfully reflect fractional ligand binding or receptor ‘activation’. (TIF) [file pone.0111314.s002.tif]

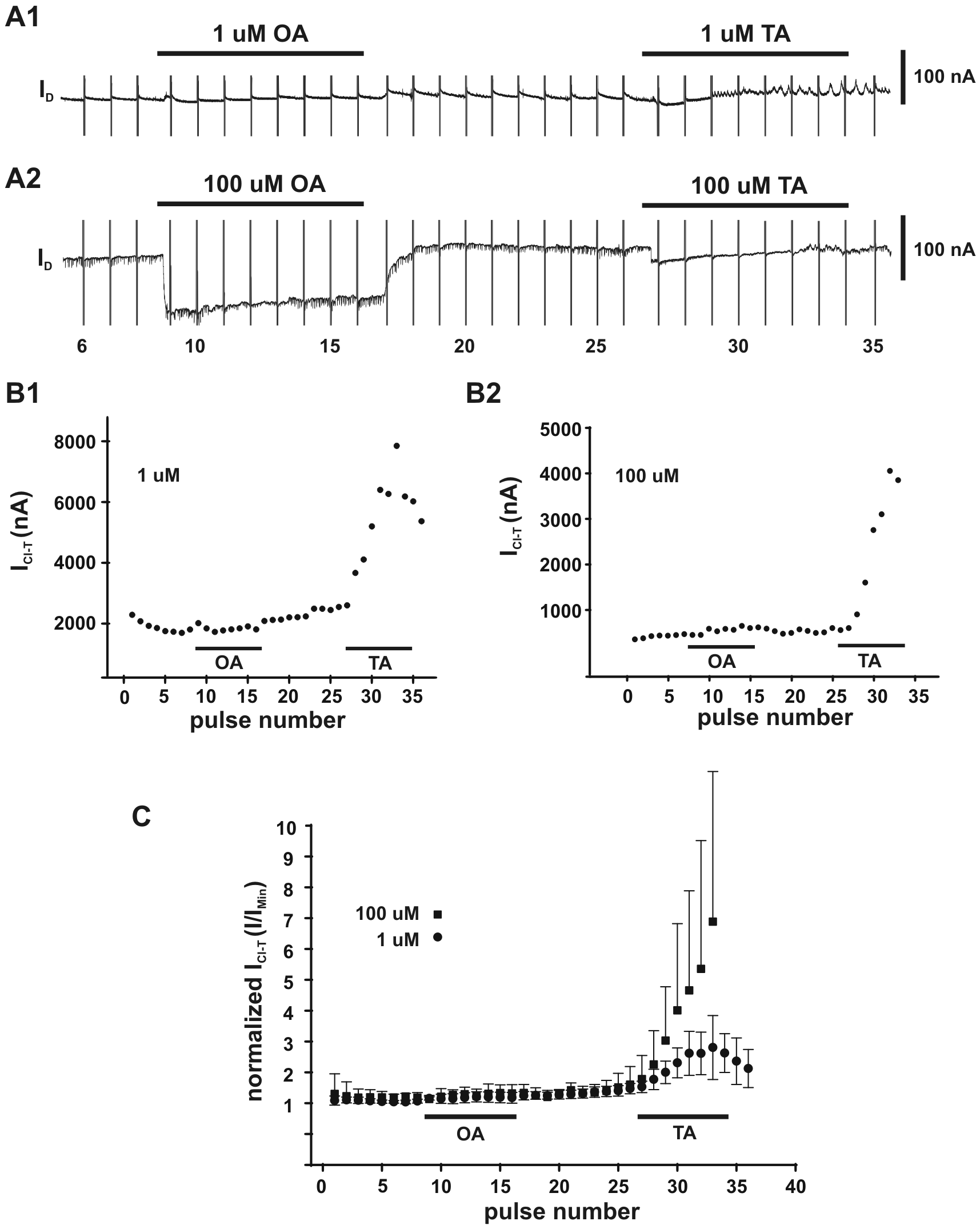

Supplement: Figure S3 — The effect of tyramine (TA) and octopamine (OA) on ICl-T at 1 µM and 100 µM. (A1 and A2) Recordings of ID from two different oocytes showing 8 minute applications of biogenic amines (black bars). The oocytes are voltage clamped at −20 mV and a measurement of ICl-T was taken every minute. The pulses used to measure ICl-T appear as vertical lines and are numbered. (B1 and B2) Individual measurements of ICl-T from the corresponding traces in A1 and A2. (C) The mean responses from 5 different oocytes at each concentration. Values for each pulse are normalized to the smallest amplitude pulse during the first 8 minutes. Error bars represent the standard deviation of the normalized values and are in one direction for 100 µM responses for clarity. (TIF) [file pone.0111314.s003.tif]

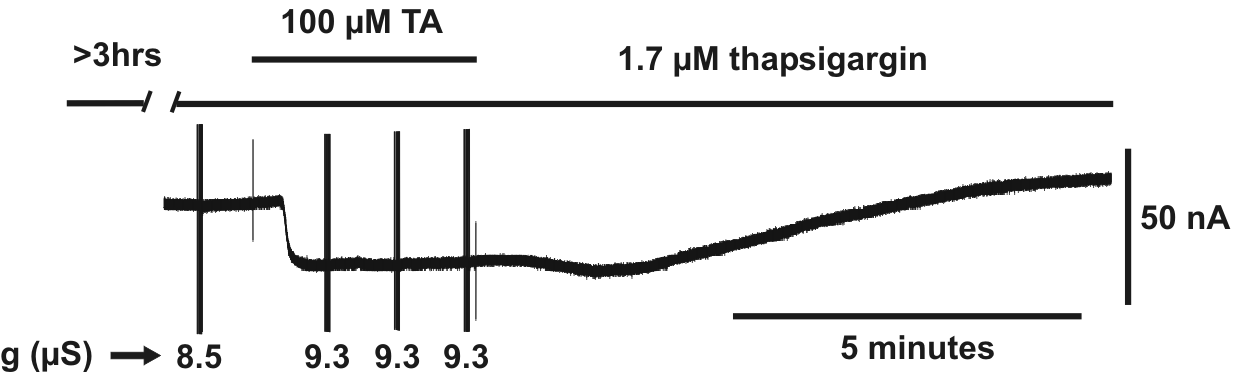

Supplement: Figure S4 — Pre-incubation in thapsigargin in calcium-free solution had no apparent effect on the tyramine induced plateau. The oscillations that were sometimes seen in normal saline (ND96) were not seen in calcium free saline. However, no obvious effects on the underlying ID waveform were seen when experiments were done in calcium-free ND96, indicating that extracellular calcium influx is not required for the development of the plateau. To specifically deplete calcium from endoplasmic reticular stores, oocytes were incubated in 1.7 µM thapsigargin in calcium-free ND 96 for three hours [59]. Under these conditions the response to tyramine was still observed and was of a similar waveform in that a plateau and second rise were still apparent (n = 2). Vertical lines are I/V pulse protocols used to monitor conductance. Measured values are given below. (TIF) [file pone.0111314.s004.tif]

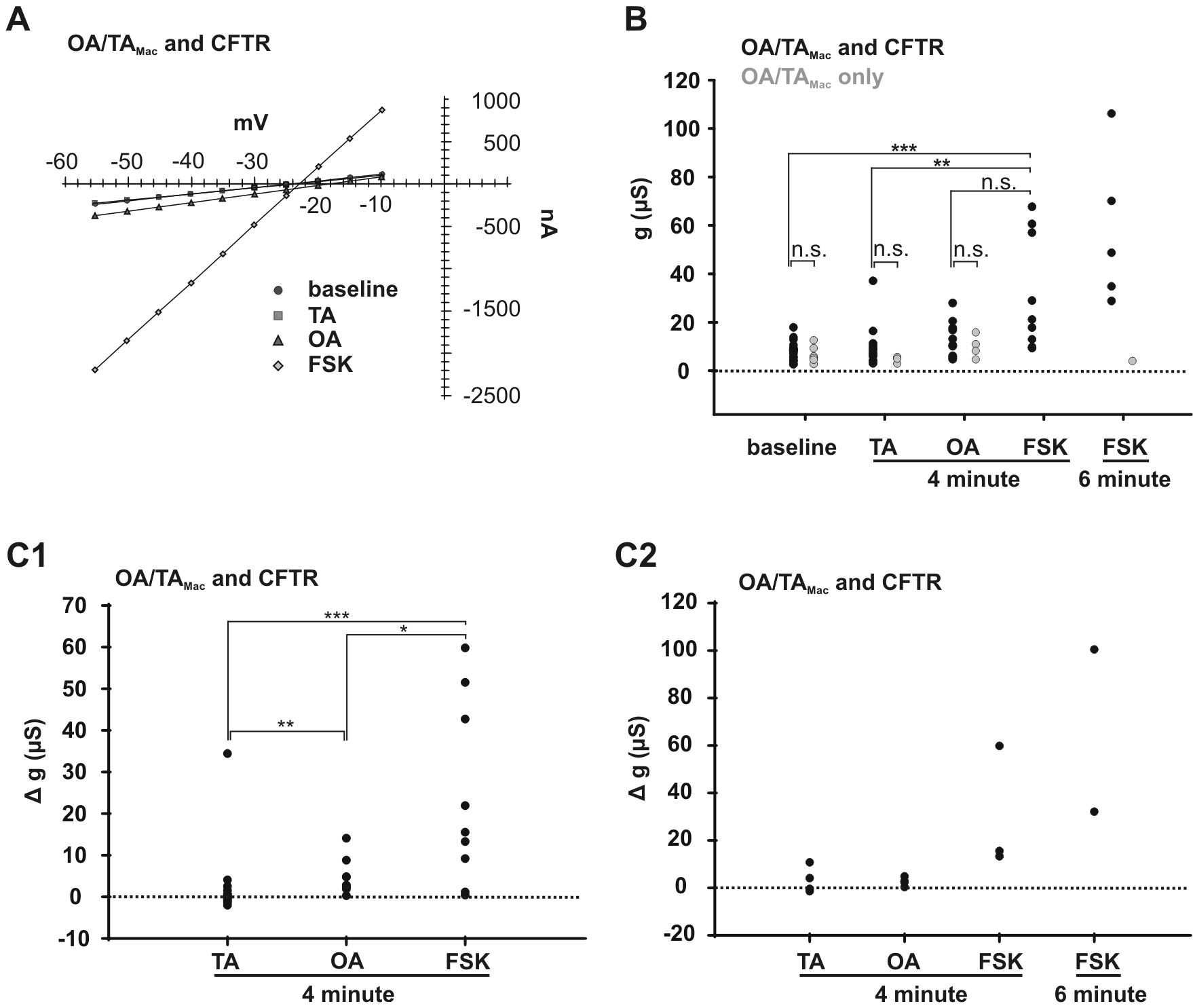

Supplement: Figure S5 — Biogenic amines do not evoke significant changes in CFTR conductance (g). (A) The measurement of whole oocyte conductance was done by plotting steady state current against the command voltage recorded during a step protocol. It is defined as the slope (I/V) of the linear least squares regression line. In the example shown tyramine (TA) causes no apparent change in whole oocyte conductance. Octopamine (OA) causes a small change compared to the adenylate cyclase activator forskolin (FSK). (B) TA or OA evoke comparable changes in conductance in oocytes injected with either OA/TAMac only, or OA/TAMac and CFTR (not significant [n.s.], p = 0.437, Mann-Whitney Rank-Sum Test). FSK evokes a significantly larger conductance than baseline or TA (**p = 0.0003) but not OA ([n.s.] p = 0.066, Mann-Whitney Rank-Sum Test). These cumulative data are from 20 OA/TAMac and CFTR-injected oocytes and 6 OA/TAMac -only injected oocytes. Conductance was measured as shown in A. Not all compounds were tested in all oocytes. Each response is treated as an independent sample. Concentrations of OA and TA were at 100 µM or 1000 µM, FSK was at 50 µM. Some points obtained at 3 minutes were grouped with the 4 minute responses. (C) The change in conductance (Δg) occurring within oocytes as determined by the difference between baseline and the indicated time points post-application of compound. The mean conductance for TA responses compiled from 12 oocytes was 0.30 µS, s.d. = 1.72 µS (high conductance outlier excluded). These values were significantly different between all responses (**TA vs OA, p = 0.002; ***TA vs FSK, p = 0.001; *OA vs FSK, p = 0.045) (Mann-Whitney Rank-Sum Test). (D) A subset of data from C showing 4 oocytes on which all three compounds were tested. The within oocyte comparison is the most appropriate and informative because it minimizes the experimental error. (TIF) [file pone.0111314.s005.tif]

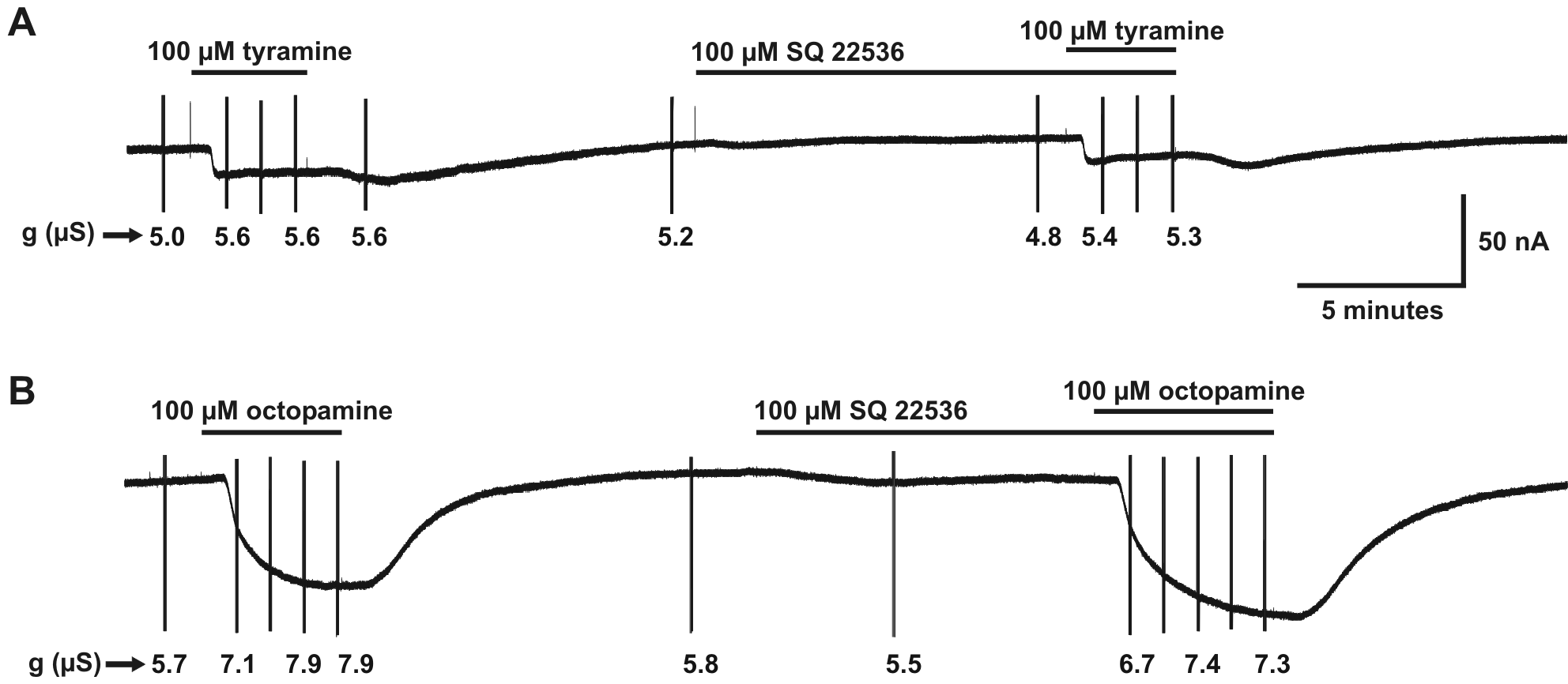

Supplement: Figure S6 — The adenylate cyclase blocker SQ-22536 produces no obvious effect on amine evoked direct-current (ID) or conductance. (A) Tyramine (TA) (n = 3). (B) Octopamine (OA) (n = 6). Vertical lines are I/V pulse protocols used to monitor conductance. Measured values are given below each vertical line. (TIF) [file pone.0111314.s006.tif]

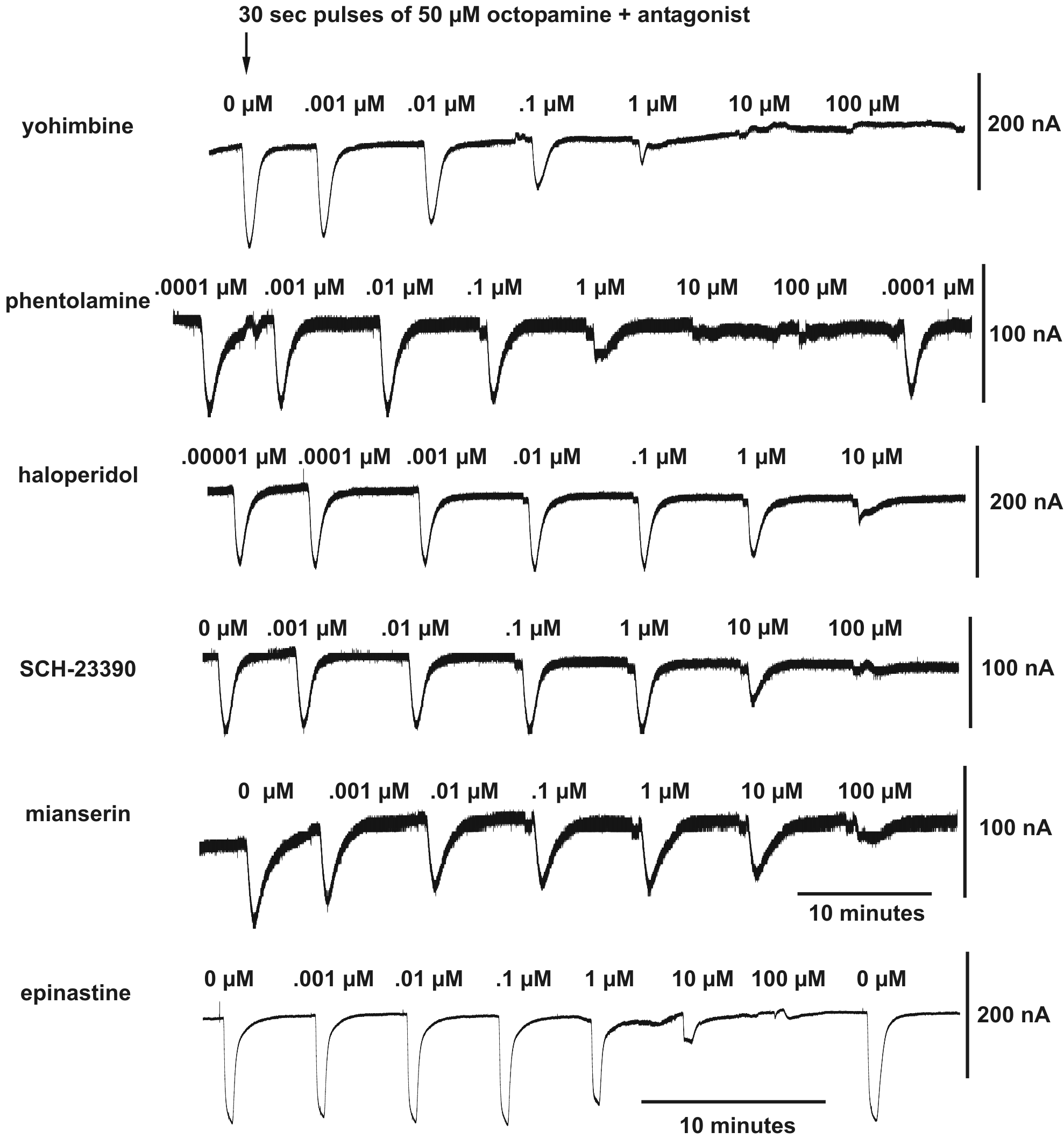

Supplement: Figure S7 — Example current traces from experiments testing putative antagonists and used to generate Figure 5B . Amplitudes plotted in Fig. 5B were normalized to the amplitudes at lowest concentration. Antagonists were applied as a mixture with 50 µM octopamine. All applications were for 30 seconds. (TIF) [file pone.0111314.s007.tif]

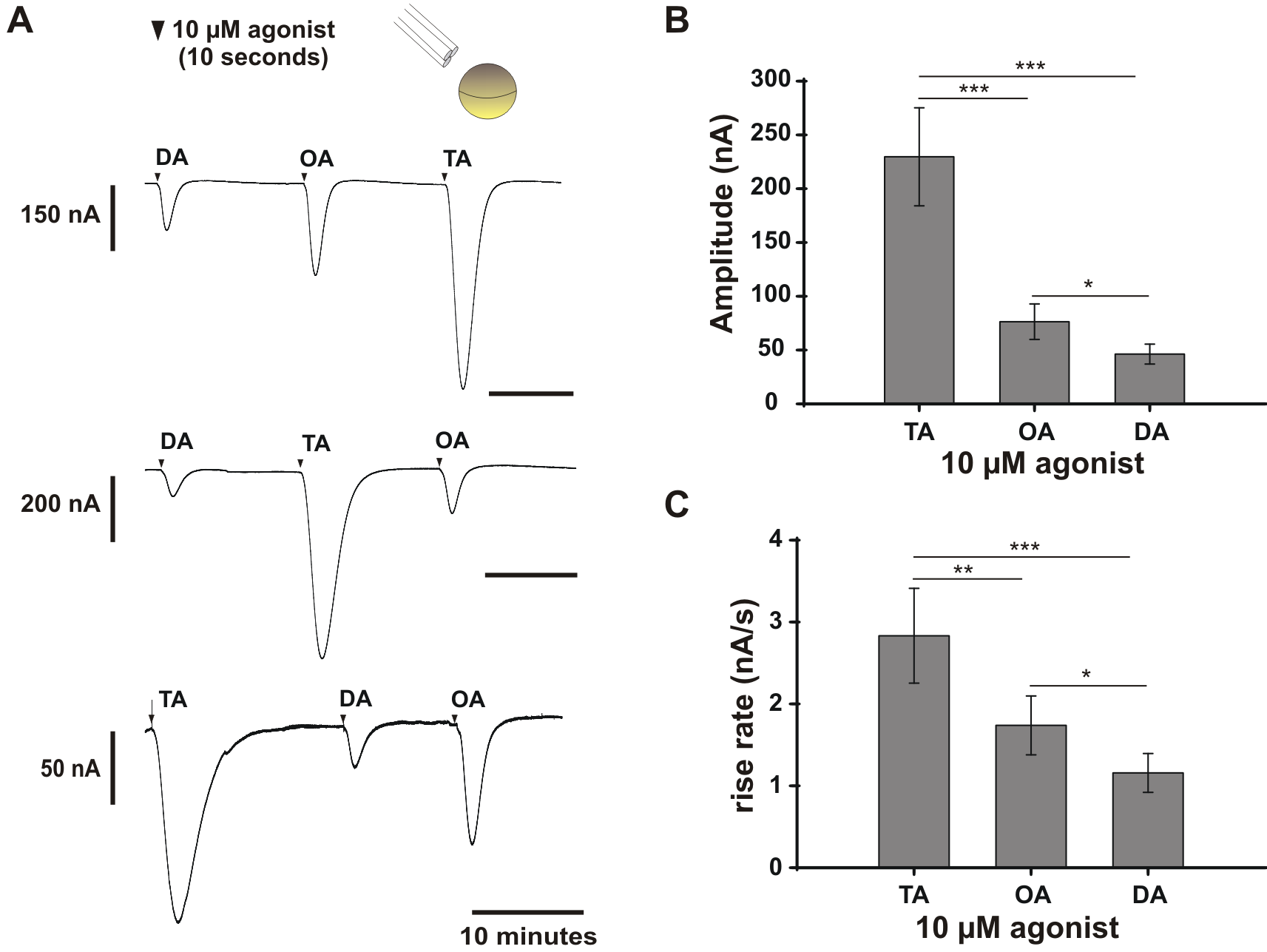

Supplement: Figure S8 — At 10 µM tyramine (TA) is more effective at evoking a direct-current (ID) response than octopamine (OA) or dopamine (DA) within single oocytes. Transmitters were applied in various orders using a 10 second focal applications indicated by arrow heads (schematic at top). The TA response was variable between oocytes (229±152 nA) and ranged from 96 nA to 563 nA. The OA (75±55 nA) and DA (46±31 nA) responses were correspondingly variable (mean ± s.d.) (n = 11 oocytes). (A) Despite between cell variability the TA response was invariably the largest within single oocytes. Representative current traces from three different oocytes are shown. (B) The mean response amplitude recorded as in A (***TA vs [OA or DA], p = <0.0001; *DA vs OA, p = 0.042). (C) The mean rise rate from the same responses shown in A and B (**TA vs OA, p = 0.002; ***TA vs DA, p = <0.001; *DA vs OA, p = 0.032). The rise rate was calculated by fitting a line to the linear portion of the rise. Data in B and C were treated as paired comparisons within single oocytes using Wilcoxon's signed-rank test. Error bars in B and C represent standard error of the mean. Oocytes were co-injected with OA/TAMac receptor and CFTR cRNA and voltage clamped at −60 mV. (TIF) [file pone.0111314.s008.tif]

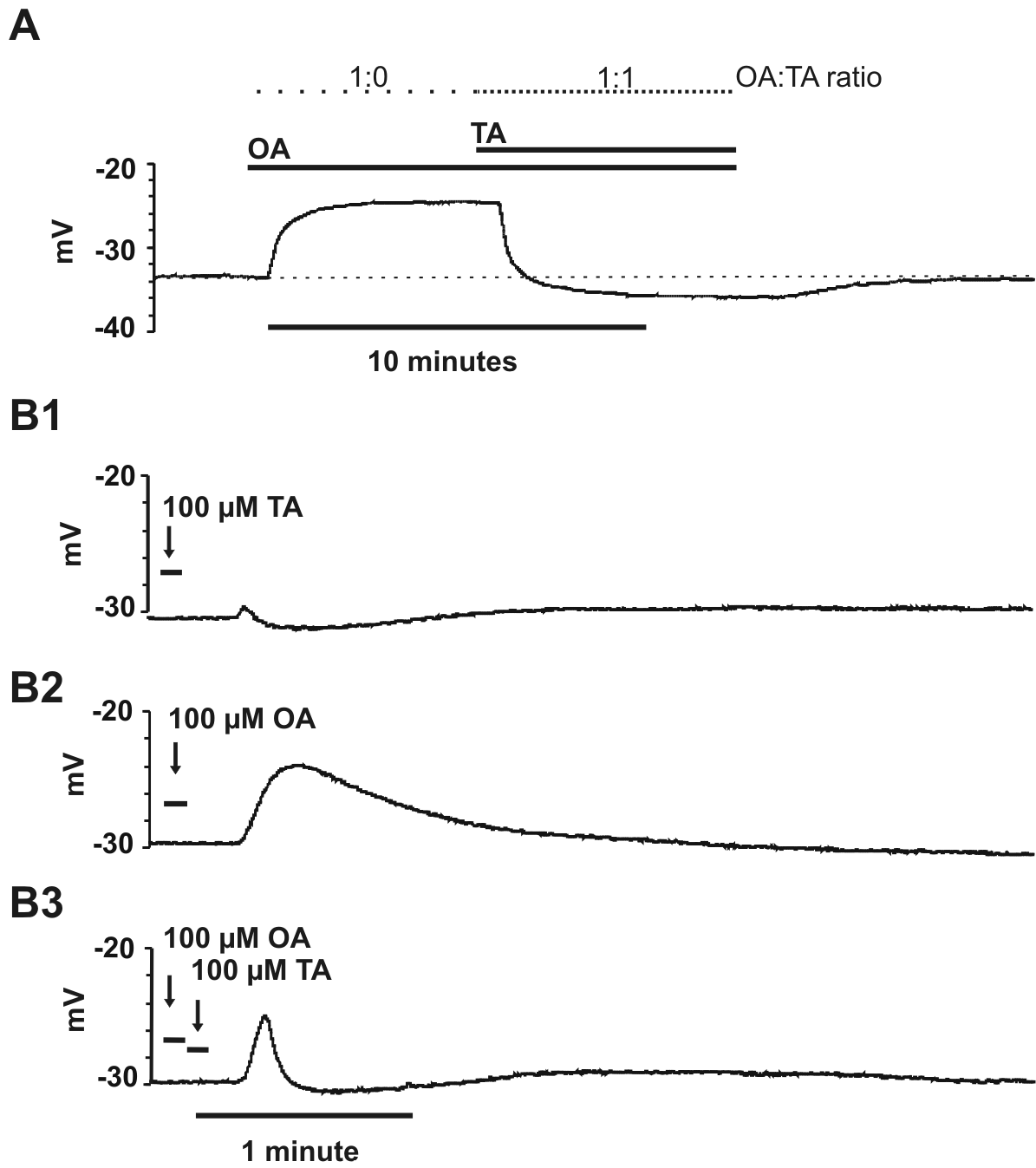

Supplement: Figure S9 — Dual exposure to octopamine (OA) and tyramine (TA) can produce variable wave-forms based on arbitrary pattering. The ratio and timing of OA and TA can modulate the membrane potential across a range. (A) At high concentration TA can induce an opposite and dominant effect to OA on membrane potential. During a long application of 100 µM OA the membrane potential appears to reach a stable level. After switching to an equally concentrated mixture of 100 µM OA and 100 µM TA the potential is reduced below baseline. (B1) A 5 second pulse of TA alone results in a bi-phasic response with a low amplitude peak. (B2) A 5 second pulse of OA alone produces a larger amplitude mono-phasic response that is clearly different. (B3) Arbitrary patterning of these applications can produce other wave-forms. (TIF) [file pone.0111314.s009.tif]
