# Supplementary figures and images for: Correction: Characterization of a Prawn OA/TA Receptor in Xenopus Oocytes Suggests Functional Selectivity between Octopamine and Tyramine
Source: PLoS One. 2015 Mar 6;10(3):e0118326. doi: 10.1371/journal.pone.0118326 (PMC4352014; doi:10.1371/journal.pone.0118326)

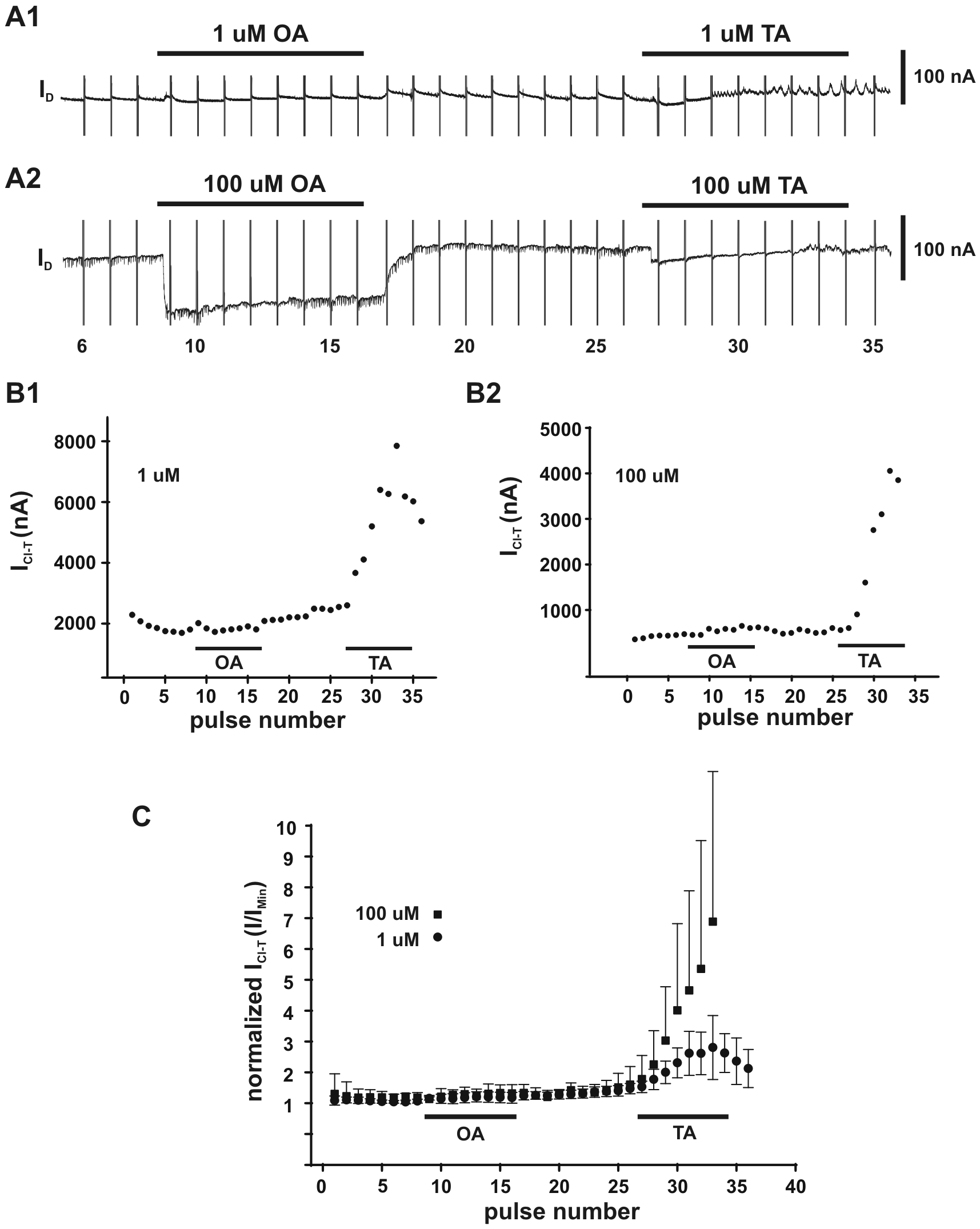

Supplement: S3 Fig — Recordings of ID from two different oocytes showing 8 minute applications of biogenic amines (black bars). The oocytes are voltage clamped at −20 mV and a measurement of ICl-T was taken every minute. The pulses used to measure ICl-T appear as vertical lines and are numbered. (B1 and B2) Individual measurements of ICl-T from the corresponding traces in A1 and A2. (C) The mean responses from 5 different oocytes at each concentration. Values for each pulse are normalized to the smallest amplitude pulse during the first 8 minutes. Error bars represent the standard deviation of the normalized values and are in one direction for 100 μM responses for clarity. (TIF) [file pone.0118326.s001.tif]

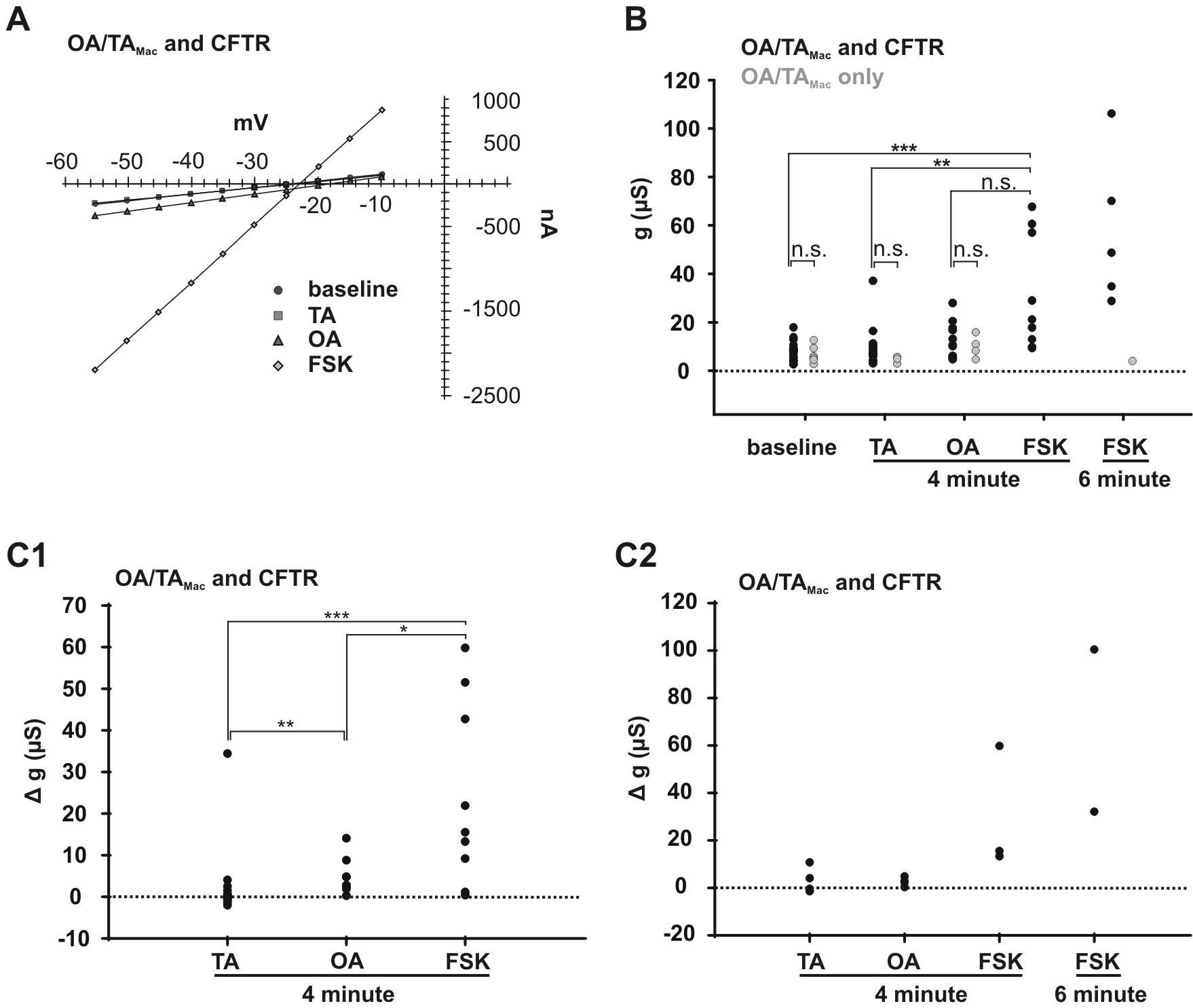

Supplement: S5 Fig — (A) The measurement of whole oocyte conductance was done by plotting steady state current against the command voltage recorded during a step protocol. It is defined as the slope (I/V) of the linear least squares regression line. In the example shown tyramine (TA) causes no apparent change in whole oocyte conductance. Octopamine (OA) causes a small change compared to the adenylate cyclase activator forskolin (FSK). (B) TA or OA evoke comparable changes in conductance in oocytes injected with either OA/TAMac only, or OA/TAMac and CFTR (not significant [n.s.], p = 0.437, Mann-Whitney Rank-Sum Test). FSK evokes a significantly larger conductance than baseline or TA (**p = 0.0003) but not OA ([n.s.] p = 0.066, Mann-Whitney Rank-Sum Test). These cumulative data are from 20 OA/TAMac and CFTR-injected oocytes and 6 OA/TAMac -only injected oocytes. Conductance was measured as shown in A. Not all compounds were tested in all oocytes. Each response is treated as an independent sample. Concentrations of OA and TA were at 100 μM or 1000 μM, FSK was at 50 μM. Some points obtained at 3 minutes were grouped with the 4 minute responses. (C) The change in conductance (Δg) occurring within oocytes as determined by the difference between baseline and the indicated time points post-application of compound. The mean conductance for TA responses compiled from 12 oocytes was 0.30 μS, s.d. = 1.72 μS (high conductance outlier excluded). These values were significantly different between all responses (**TA vs OA, p = 0.002; ***TA vs FSK, p = 0.001; *OA vs FSK, p = 0.045) (Mann-Whitney Rank-Sum Test). (D) A subset of data from C showing 4 oocytes on which all three compounds were tested. The within oocyte comparison is the most appropriate and informative because it minimizes the experimental error. (TIF) [file pone.0118326.s002.tif]

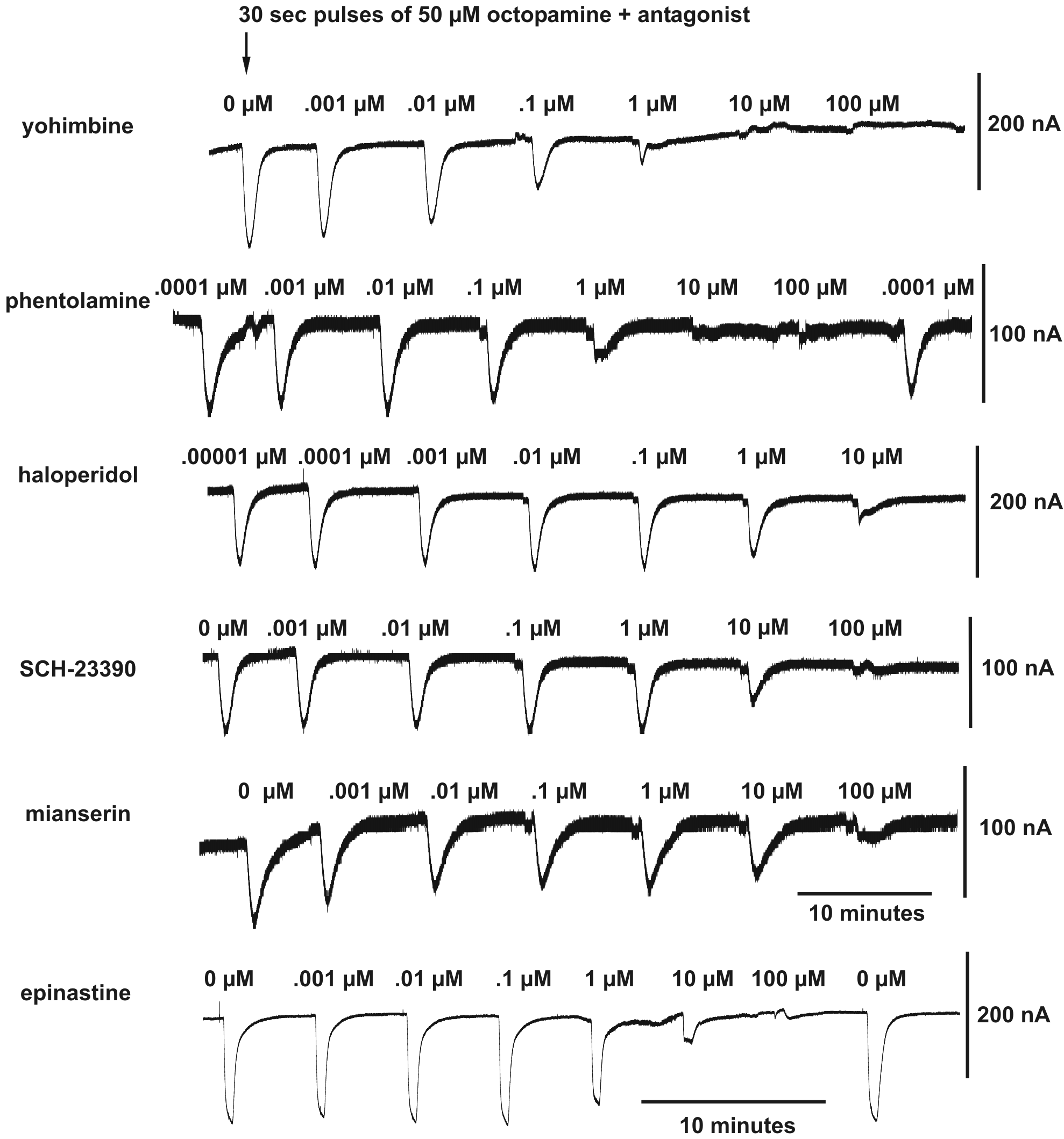

Supplement: S7 Fig — Amplitudes plotted in Fig. 5B were normalized to the amplitudes at lowest concentration. Antagonists were applied as a mixture with 50 μM octopamine. All applications were for 30 seconds. (TIF) [file pone.0118326.s003.tif]

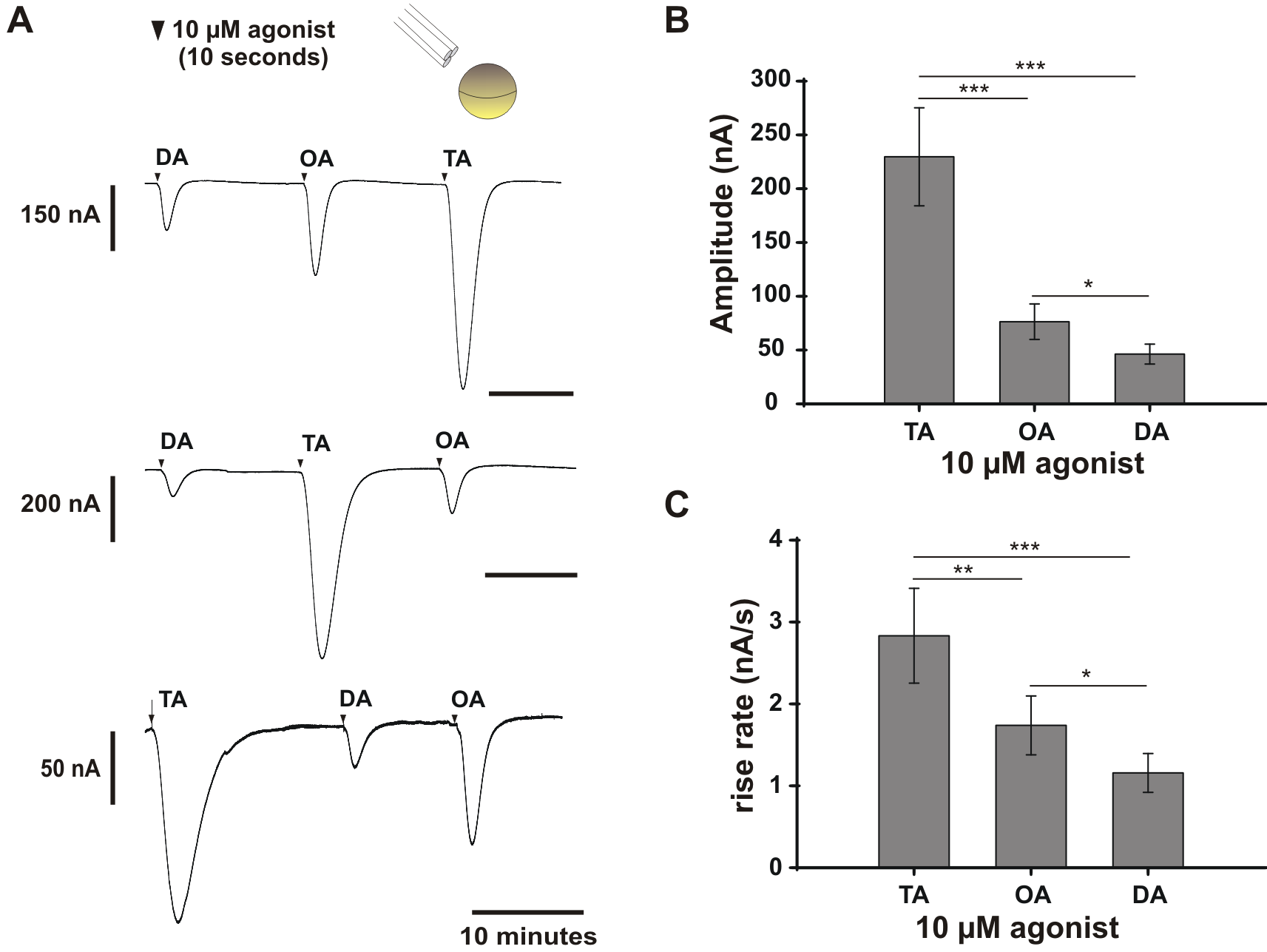

Supplement: S8 Fig — Transmitters were applied in various orders using a 10 second focal applications indicated by arrow heads (schematic at top). The TA response was variable between oocytes (229±152 nA) and ranged from 96 nA to 563 nA. The OA (75±55 nA) and DA (46±31 nA) responses were correspondingly variable (mean ± s.d.) (n = 11 oocytes). (A) Despite between cell variability the TA response was invariably the largest within single oocytes. Representative current traces from three different oocytes are shown. (B) The mean response amplitude recorded as in A (***TA vs [OA or DA], p = <0.0001; *DA vs OA, p = 0.042). (C) The mean rise rate from the same responses shown in A and B (**TA vs OA, p = 0.002; ***TA vs DA, p = <0.001; *DA vs OA, p = 0.032). The rise rate was calculated by fitting a line to the linear portion of the rise. Data in B and C were treated as paired comparisons within single oocytes using Wilcoxon's signed-rank test. Error bars in B and C represent standard error of the mean. Oocytes were co-injected with OA/TAMac receptor and CFTR cRNA and voltage clamped at −60 mV. (TIF) [file pone.0118326.s004.tif]
